# Supplementary material for: Aspartyl proteases target host actin nucleator complex protein to limit epithelial innate immunity
Source: EMBO Rep. 2024 Sep 30;25(11):4846–75. doi: 10.1038/s44319-024-00270-y (PMC11549443; doi:10.1038/s44319-024-00270-y)
Supplement: Supplementary file 1 — Appendix [file 44319_2024_270_MOESM1_ESM.pdf]

Appendix

Table of Contents

Appendix Figure S1……………………………………………………………… Page 2

Appendix Figure S2……………………………………………………………. Page 3

Appendix Table S1………………………………………………………………Page 5

Appendix Table S2………………………………………………………………Page 8

Appendix Figure S1

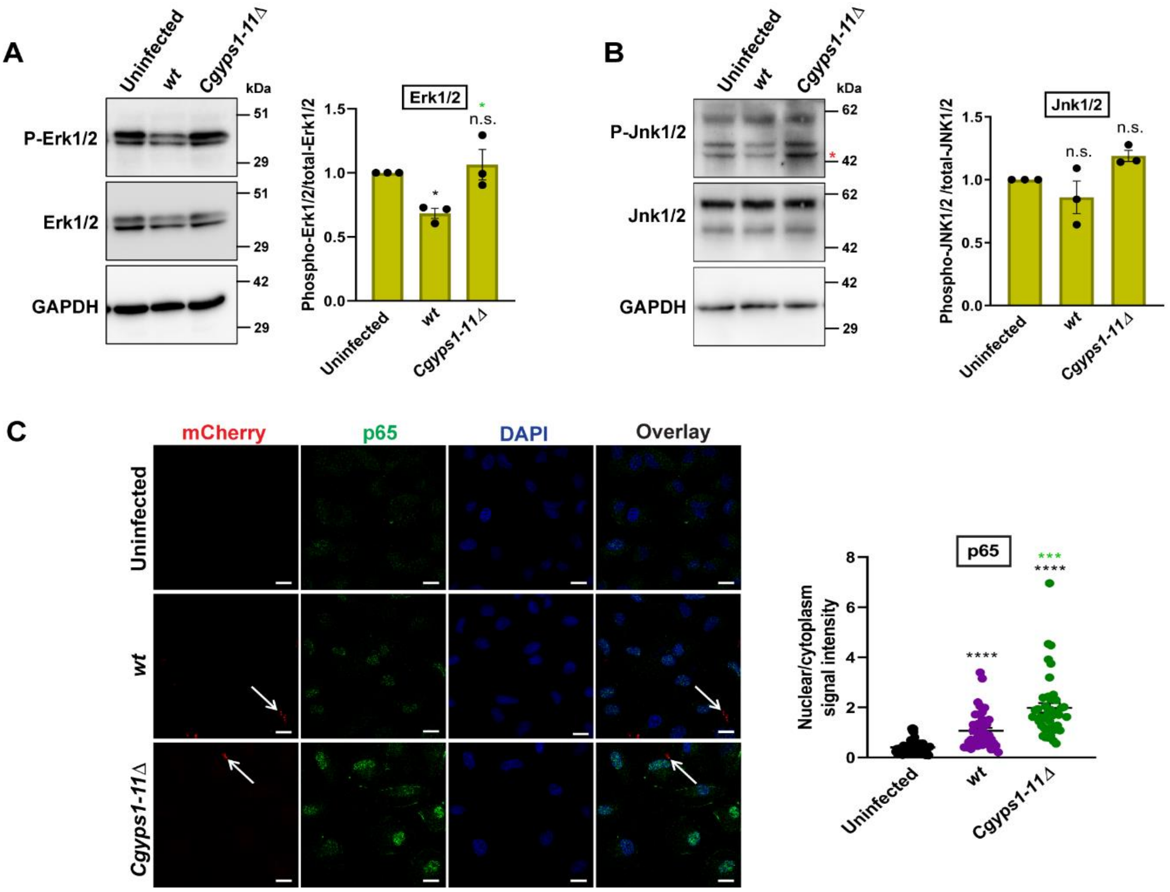

Appendix Figure S1: *Cgyps1-11*Δ infection to A-498 cells leads to elevated nuclear localization of p65.

A & B. Representative immunoblots illustrating phosphorylated-Erk1/2 (**A**) and phosphorylated-Jnk1/2 (**B**) levels in indicated A-498 cells after 6 h of *Cg* infection. The red asterisk denotes non-specific band. *n*=3 biological replicates. Data represent fold-change in phosphorylated-Erk1/2 (**A**) and phosphorylated-Jnk1/2 (**B**) levels in *Cg*-infected A-498 cells, compared to uninfected A-498 cells (considered as 1.0). Green and black asterisks indicate statistically-significant differences, compared to *wt*-infected and uninfected A-498 cells, respectively.

C. Confocal micrographs illustrating the cellular localization of p65 in indicated of A-498 cells after 6 h of *Cg* infection. Immunofluorescence analysis of A-498 cells was performed with anti-p65 antibody, and images were captured using the confocal microscope (Leica SP8) with 63X/1.44 NA objective lens in the z-stack mode. White arrows mark mCherry-expressing *Cg*. Fluorescence signal intensities in the cytoplasm and the nucleus were measured in a minimum of 40 cells, using the ImageJ software. *n*=3 biological replicates. Data are plotted on the right side of micrographs. Green and black asterisks indicate statistically-significant differences, as compared to *wt*-infected and uninfected A-498 cells, respectively.

Data information: In (**A, B, C**), data are presented as mean ± SEM. *$P < 0.05$; ***$P < 0.001$; ****$P < 0.0001$; n.s, not significant. Unpaired or paired two-tailed Student's t test. $P = 0.0151$

(*wt* vs. uninfected), *P* = 0.0379 (*Cgyps1-11Δ* vs. *wt*) in (**A**). *P* = 0.00000063 (*wt* vs. uninfected), *P* = 0.000000000004 (*Cgyps1-11Δ* vs. uninfected), *P* = 0.0001 (*Cgyps1-11Δ* vs. *wt*) in (**C**). Scale bar = 20 µm in (**C**).

Appendix Figure S2

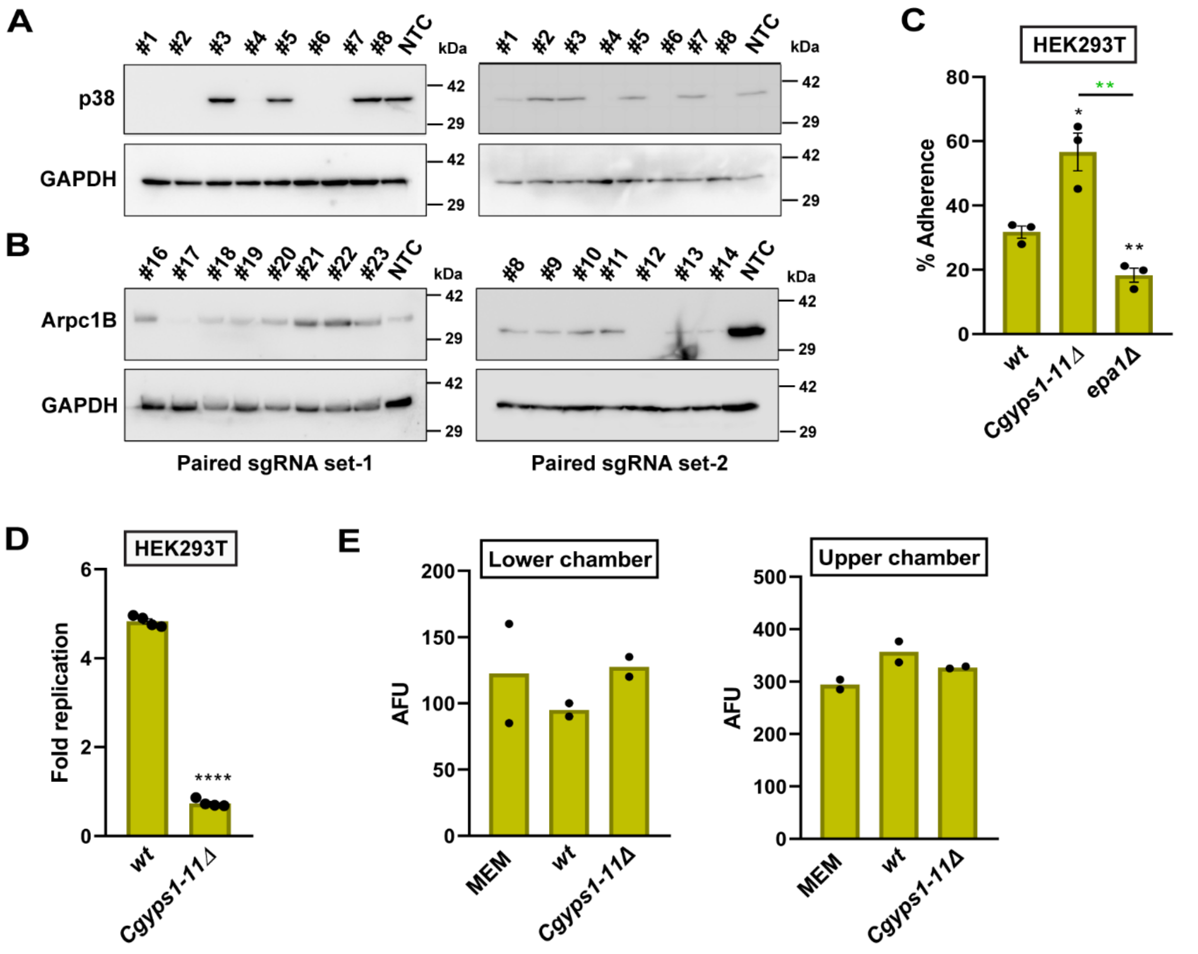

Appendix Figure S2: *Cg* adheres to and replicates in HEK293T cells.

A. Representative immunoblots verifying p38 knockout in generated *p38*$^{-/-}$ HEK293T cells. Lysates (120 µg) of *p38*$^{-/-}$ HEK293T cells, created by two sets of paired guide-RNAs (Set1 ad Set2), were resolved on 12% SDS-PAGE and probed with anti-p38 and anti-GAPDH antibodies. Clones #6 and #8 for Set1 and Set2 sgRNA, respectively, were selected for analysis.

B. Representative immunoblots verifying Arpc1B knockout in generated *Arpc1B*$^{-/-}$ HEK293T cells. Lysates (120 µg) of *Arpc1B*$^{-/-}$ HEK293T cells, created by two sets of paired guide-RNAs (Set1 ad Set2), were resolved on 12% SDS-PAGE and probed with Arpc1B and anti-GAPDH antibodies. Clones #17 and #12 for Set1 and Set2 sgRNA, respectively, were selected for analysis.

C. Adherence of indicated *Cg* strains to fixed HEK293T cells after 2 h co-incubation as measured by CFU-based assay. *n*=3 biological replicates. Black asterisks denote statistically-significant adherence differences, compared to *wt*-infected HEK293T cells.

D. Intracellular *Cg* survival analysis in HEK293T epithelial cells. Fold replication represents the ratio of 24 h CFUs to 4 h CFUs. *n*=4 biological replicates.

E. Migration of FITC-conjugated anti-CD66b-labelled human neutrophils towards *wt* and *Cgyps1-11*Δ strains, as measured by recording fluorescence of the neutrophils present in the upper and lower chamber of the transwell filter. YPD-grown *Cg* strains were washed with PBS, and placed (1 x10^6 cells in MEM medium) in the lower chamber. Isolated neutrophils (1 x10^6 cells in MEM medium) were added to the upper chamber. After *Cg*-neutrophil co-culturing for 2 h at 37°C, neutrophil migration towards *Cg* strains was recorded by measuring the fluorescence of neutrophils in both chambers. Data (mean; *n*=2 biological replicates) are expressed as Arbitrary Fluorescent Unit (AFU).

Data information: In (**C, D**), data are presented as mean ± SEM. **P* < 0.05; ***P* < 0.01; *****P* < 0.0001; Unpaired two-tailed Student's t test. *P* = 0.0157 (*Cgyps1-11Δ* vs. *wt*), *P* = 0.0099 (*epa1Δ* vs. *wt*), *P* = 0.0036 (*Cgyps1-11Δ* vs. *epa1Δ*) in (**C**). *P* = 0.000000002 (*Cgyps1-11Δ* vs. *wt*) in (**D**).

Appendix Table S1: List of *Candida glabrata* strains (A) and plasmids (B) used in the study

Appendix Table S1A: List of *C. glabrata* strains used in the study.

Yeast strain	Genotype	Reference
YRK19	*ura3Δ::Tn903 G418R* (BG14)	Cormack and Falkow, 1999
YRK20	*URA3* (BG462)	De Las Peñas *et al*., 2003
YRK1091	*ura3Δ::Tn903 G418R Cgyps1Δ::hph*	Kaur *et al*., 2007
YRK1996	*ura3Δ::Tn903 G418R Cgyps7Δ::hph*	Kaur *et al*., 2007
YRK1002	*URA3 Cgyps1Δ::hph*	Kaur *et al*., 2007
YRK1003	*URA3 Cgyps7Δ::hph*	Kaur *et al*., 2007
YRK1005	*URA3 Cgyps2ΔCΔ::hph*	Kaur *et al*., 2007
YRK1997	*ura3Δ::Tn903 G418R Cgyps(1-11)Δ::hph*	Kaur *et al*., 2007
YRK3002	*URA3 Cgyps(1-11)Δ::hph*	Kaur *et al*., 2007
YRK1123	*URA3 epa1Δ::hph*	Rai *et al*., 2015
YRK5266	*ura3Δ::Tn903 G418R epa1Δ::hph*	This study
YRK1125	*URA3 epa1Δ6Δ7Δ::hph*	Brendan Cormack laboratory
YRK5074	*URA3 Cgyps1-11Δepa1Δ::nat1*	Kumar *et al*., 2024
YRK1299	*ura3Δ::Tn903 G418R* (YRK19)*/pRK999*	Kumar *et al*., 2024
YRK3221	*ura3Δ::Tn903 G418R Cgyps1Δ::hph (YRK1091)/pRK1462*	Battu *et al*., 2021
YRK1304	*ura3Δ::Tn903 G418R Cgyps7Δ::hph (YRK1996)/pRK1051*	Kaur Laboratory
YRK3223	*ura3Δ::Tn903 G418R Cgyps1Δ::hph (YRK1091)/pRK1039*	Kaur Laboratory
YRK4805	*ura3Δ::Tn903 G418R Cgyps1Δ::hph (YRK1091)/pRK2312*	This study
YRK2552	*ura3Δ::Tn903 G418R Cgyps1-11Δ::hph (YRK1997)/pRK999*	This study
YRK4453	*ura3Δ::Tn903 G418R Cgyps1-11Δ::hph (YRK1997)/pRK1462*	This study
YRK4455	*ura3Δ::Tn903 G418R Cgyps1-11Δ::hph (YRK1997)/pRK1051*	This study
YRK4879	*ura3Δ::Tn903 G418R Cgyps1-11Δ::hph (YRK1997)/pRK1039*	This study
YRK4807	*ura3Δ::Tn903 G418R Cgyps1-11Δ::hph (YRK1997)/pRK2312*	This study
YRK4313	*ura3Δ::Tn903 G418R* (YRK19)*/pRK1315*	This study
YRK4324	*ura3Δ::Tn903 G418R Cgyps1-11Δ::hph (YRK1997)/pRK1315*	This study
YRK5274	*ura3Δ::Tn903 G418R epa1Δ::hph (YRK5266)/pRK1315*	This study
YRK29	*Candida albicans* SC5314	Kaur Laboratory
YRK1845	*CgYPS1* + pPIC-9 linearised with SacI and transformed into GS115 strain (*Pichia pastoris*) (His$^+$Mut^S)	Battu *et al*., 2021

YRK3083	*CgYPS1*^{D91A} + pPIC-9 linearised with SacI and transformed into GS115 strain (*Pichia pastoris*) ($His^{+}Mut^{S}$)	Battu *et al.,* 2021

References

Cormack BP, Falkow S. (1999) Efficient homologous and illegitimate recombination in the opportunistic yeast pathogen *Candida glabrata*. *Genetics* **151**:979-87.

De Las Penas A, *et al*. (2003) Virulence-related surface glycoproteins in the yeast pathogen *Candida glabrata* are encoded in subtelomeric clusters and subject to RAP1- and SIR-dependent transcriptional silencing. *Genes Dev* **17**:2245-58

Kaur R, Ma B, Cormack BP (2007) A family of glycosylphosphatidylinositol-linked aspartyl proteases is required for virulence of *Candida glabrata*. *Proc Natl Acad Sci USA* **104:**7628-33.

Rai MN, Sharma V, Balusu S, Kaur R. (2015) An essential role for phosphatidylinositol 3-kinase in the inhibition of phagosomal maturation, intracellular survival and virulence in *Candida glabrata*. *Cell Microbiol* **17**:269–87

Kumar K, Pareek A, Kaur R (2024) SWI/SNF complex-mediated chromatin remodelling is vital for cell surface adhesin repression and immune evasion. *iScience* **27**:109607

Battu A, Purushotham R, Dey P, Vamshi SS, Kaur R (2021) An aspartyl protease-mediated cleavage regulates structure and function of a flavodoxin-like protein and aids oxidative stress survival. *PLoS Pathog* **17**: e1009355.

Appendix Table S1B: List of plasmids used in the study.

Plasmid	Description	Reference
pRK999	pCU-PDC1 plasmid	Addgene (Plasmid #45323)
pRK1315	pYC34-mCherry plasmid	Addgene (Plasmid #63915)
pRK1462	*CgYPS1* (1.5 kb) cloned in *SpeI-XmaI* sites of pRK999 plasmid	Battu *et al.,* 2021
pRK1051	*CgYPS7* (1.7 kb) cloned in *XbaI-XhoI* sites of pRK999 plasmid	Askari *et al.,* 2022
pRK1039	*CgYPS1*^{D91A}(1.5 kb) cloned in *Spe1-XmaI* sites of pRK999 plasmid	Battu *et al.,* 2021
pRK2312	*CgYPS1*^{D378A} (1.5 kb) cloned in *Spe1-XmaI* sites of pRK999 plasmid	This study
pRK2306	pU6-Cas9 plasmid for CRISPR-Cas9 knock out	P ChandraShekar Laboratory, CSIR-CCMB, Hyderabad, India
pRK2397	*sgRNA3* (for p38 KO) cloned in pU6-Cas9-GFP plasmid at BbsI site	This study
pRK2370	*sgRNA4* (for p38 KO) cloned in pU6-Cas9-GFP plasmid at BbsI site	This study
pRK2384	*sgRNA6* (for p38 KO) cloned in pU6-Cas9-GFP plasmid at BbsI site	This study
pRK2478	*sgRNA1* (for Arpc1B KO) cloned in pU6-Cas9-GFP plasmid at BbsI site	This study
pRK2372	*sgRNA2* (for Arpc1B KO) cloned in pU6-Cas9-GFP plasmid at BbsI site	This study
pRK2544	*sgRNA5* (for Arpc1B KO) cloned in pU6-Cas9-GFP plasmid at BbsI site	This study

pRK2546	*sgRNA6* (for Arpc1B KO) cloned in pU6-Cas9-GFP plasmid at BbsI site	This study
pRK2304	pDONR201 Donor Gateway plasmid	M. S. Reddy Laboratory, BRIC-CDFD, Hyderabad, India
pRK2326	Gateway destination plasmid (C-terminal SFB)	M. S. Reddy Laboratory, BRIC-CDFD, Hyderabad, India
pRK2380	GFP-SFB destination plasmid	M. S. Reddy Laboratory, BRIC-CDFD, Hyderabad, India
pRK2308	*Arpc1B* gene cloned in pDONR201 plasmid at attP and attB sites	This study
pRK2354	*Arpc1B* gene cloned in destination plasmid at attL and attR sites	This study
pRK2694	*Arpc1B*(R142A) gene cloned in destination plasmid at attL and attR sites	This study
pRK2702	*Arpc1B*(R74A) gene cloned in destination plasmid at attL and attR sites	This study

References

Battu A, Purushotham R, Dey P, Vamshi SS, Kaur R (2021) An aspartyl protease-mediated cleavage regulates structure and function of a flavodoxin-like protein and aids oxidative stress survival. *PLoS Pathog* **17**: e1009355.

Askari F, Rasheed M, Kaur R (2022) The yapsin family of aspartyl proteases regulate glucose homeostasis in *Candida glabrata*. *J Biol Chem 298*:101593.

Appendix Table S2: List of primers (A), antibodies (B), inhibitors (C) and cytokines (D) used in the study

Appendix Table S2A: List of primers used in the study.

Primer	Sequence (5'-3')	Description	Restriction enzyme	Reference
**For generation of knockout cell lines**				
OgRK 4653	CACCGTGGATGCATTAC AACCAGAC	p38 CRISPR sgRNA For	BbsI	This study
OgRK 4654	AAACGTCTGGTTGTAAT GCATCCAC	p38 CRISPR sgRNA Rev	BbsI	This study
OgRK 4655	CACCGTCCTCGGGACAT GGTGGATC	p38 CRISPR sgRNA For	BbsI	This study
OgRK 4656	AAACGATCCACCATGTCC CGAGGAC	p38 CRISPR sgRNA Rev	BbsI	This study
OgRK 4657	CACCGTAGGTCAAGTCG TACTAGAG	p38 CRISPR sgRNA For	BbsI	This study
OgRK 4658	AAACCTCTAGTACGACTT GACCTAC	p38 CRISPR sgRNA Rev	BbsI	This study
OgRK 4892	CACCGTTCGAATCCAGCA GTAGCTG	ARPC1B CRISPR sgRNA For	BbsI	This study
OgRK 4893	AAACCAGCTACTGCTGG ATTCGAAC	ARPC1B CRISPR sgRNA Rev	BbsI	This study
OgRK 4894	CACCGTTCCCGTAGAACC TCGGGGT	ARPC1B CRISPR sgRNA For	BbsI	This study
OgRK 4895	AAACACCCCGAGGTTCT ACGGGAAC	ARPC1B CRISPR sgRNA Rev	BbsI	This study
OgRK 4900	CACCGGGCTCCAAGATG CCCTTTG	ARPC1B CRISPR sgRNA For	BbsI	This study
OgRK 4901	AAACCAAAGGGCATCTT GGAGCCC	ARPC1B CRISPR sgRNA Rev	BbsI	This study
OgRK 4902	CACCGAAGGAGGTGGA GGAACTACA	ARPC1B CRISPR sgRNA For	BbsI	This study
OgRK 4903	AAACTGTAGTTCCTCCAC CTCCTTC	ARPC1B CRISPR sgRNA Rev	BbsI	This study
**For gene cloning**				
OgRK 4618	GGGGACAAGTTTGTACA AAAAAGCAGGCTTCATG GCCTACCACAGCTTC	*Arpc1B*_Forward_ Gateway cloning		This study
OgRK 4619	GGGGACCACTTTGTACA AGAAAGCTGGGTTTTTG ATCTTGAGGTCCTTC	*Arpc1B*_Reverse_ Gateway cloning		This study
OgRK 4663	ATGGCCTACCACAGCTTC CTG	*Arpc1B*_Forward_ full CDS		This study
OgRK 4664	TCATTTGATCTTGAGGTC CTT	*Arpc1B*_Reverse_ full CDS		This study
OgRK 4665	GCCCCCGAGAGTAACCG TAT	*Arpc1B*_Forward_ internal		This study
OgRK 4666	CTCGCGGGCCGTCAAGC CAC	*Arpc1B*_Reverse_ internal		This study

OgRK 4708	GGGGACCACTTTGTACAAGAAAGCTGGGTTTCATTTGATCTTGAGGTCC	*Arpc1B*_Reverse_Gateway cloning (with stop codon)	This study
**For site-directed mutagenesis**			
OgRK 4614	GATCCCCGCCCTATTGGCCTCTGGTACCACCTTAAC	*CgYPS1* (D378A)_Forward	This study
OgRK 4615	GTTAAGGTGGTACCAGAGGCCAATAGGGCGGGGATC	*CgYPS1* (D378A)_Reverse	This study
OgRK 5154	CATCAAGAAGCCCATCGCTTCCACCGTCCTCAGC	*Arpc1B* (R142A)_Forward	This study
OgRK 5155	GCTGAGGACGGTGGAAGCGATGGGCTTCTTGATG	*Arpc1B* (R142A)_Reverse	This study
OgRK 5394	ATTGTGACCTGCGGCACAGACGCAAACGCCTACGTGTGGACGCTG	*Arpc1B* (R74A)_Forward	This study
OgRK 5395	CAGCGTCCACACGTAGGCGTTTGCGTCTGTGCCGCAGGTCACAAT	*Arpc1B* (R74A)_Reverse	This study
**For qRT-PCR**			
OgRK 4888	ACGACTGCTTCCCGGTGCTG	*Arpc1B*_Forward	This study
OgRK 4889	CGCTGTTCTTGTGCAGCGAG	*Arpc1B*_Reverse	This study
OgRK1789	ACCTGCCAAATATGATGAC	*GAPDH*_Forward	Kaur Laboratory
OgRK1790	TCATACCAGGAAATGAGCTT	*GAPDH*_Reverse	Kaur Laboratory

*** The underlined sequences correspond to cleavage sites of restriction enzymes.**

Appendix Table S2B: List of antibodies and inhibitors used in the study.

**Name**	**Dilution used**	**Clonality**	**Company**	**Catalog number**
**For immunoblotting analysis**				
**Primary antibodies**				
Akt antibody	1:5000	Polyclonal	Cell Signaling Technology	9272
Phospho-Akt (Ser473) antibody	1:1000	Polyclonal	Cell Signaling Technology	9018
p38 MAPK antibody	1:5000	Monoclonal	Cell Signaling Technology	9228
Phospho-p38 MAPK antibody	1:5000	Monoclonal	Cell Signaling Technology	4511
p44/42 MAPK antibody	1:5000	Polyclonal	Cell Signaling Technology	9102

Phospho-p44/42 MAPK antibody	1:5000	Monoclonal	Cell Signaling Technology	4370
SAPK/Jnk antibody	1:5000	Polyclonal	Cell Signaling Technology	9252
phospho-SAPK/Jnk antibody	1:1000	Polyclonal	Cell Signaling Technology	9251
p65 antibody	1:5000	Monoclonal	Cell Signaling Technology	8242
Phospho-p65 antibody	1:1000	Monoclonal	Cell Signaling Technology	3033
Arpc1B antibody	1:1000	Monoclonal	Santacruz Biotechnology	SC-137125
Anti-GAPDH	1:10000	Polyclonal	Abcam	ab22555
Anti-actin	1:10000	Monoclonal	Milipore	MAB1501
Anti-Flag	1:10000	Monoclonal	Sigma	F1804
Anti-6XHis	1:6000	Polyclonal	Abcam	ab9108
**Secondary antibodies**				
Anti-rabbit IgG-HRP	1:10000		Cell Signaling Technology	7074
Anti-mouse IgG-HRP	1:10000		Cell Signaling Technology	7076
**For immunofluorescence analysis**				
**Primary antibodies**				
Anti-Flag	1:500	Monoclonal	Sigma	F1804
p65 antibody	1:500	Monoclonal	Cell Signaling Technology	8242
Arpc1B antibody	1:100	Monoclonal	Santacruz Biotechnology	SC-137125
p38 MAPK antibody	1:200	Polyclonal	Novus Biologicals	NB110-96907
**Secondary antibodies**				
FITC-conjugated anti-Human CD66b antibody	1:150	Monoclonal	BD Biosciences	555724
Alexa Fluor 488 goat anti-rabbit IgG (H+L)	1:300	Polyclonal	Abcam	ab150077
Alexa Fluor 647 goat anti-mouse IgG (H+L)	1:300	Polyclonal	Thermo scientific	A-21236
Alexa Fluor 488 goat anti-mouse IgG (H+L)	1:300	Polyclonal	Thermo scientific	A-11029
Rhodamine-Phalloidin	1:500	Stain	Thermo scientific	R415
Annexin V-Alexa Fluor 488		Stain	Thermo scientific	A13201
**For immunohistochemistry analysis**				
**Primary antibodies**				

Anti-rabbit Ly6G	1:1000	Monoclonal	Abcam	Ab238132
Anti-rabbit CD45+	1:400	Polyclonal	Abcam	ab10558
**Secondary antibodies**				
Anti-rabbit IgG-HRP	1:1000		Cell Signaling Technology	7074

Appendix Table S2C: List of inhibitors used in the study.

Name	Target	Company	Catalog number
Dynasore	Dynamin	Abcam	ab120192
BAY 11-7082	NF-kB	Sigma	B5556
SB 203580	p38 MAPK	Cell Signaling Technology	5633S
CK-666	Arp2/3 complex	Sigma	182515

Appendix Table S2D: List of cytokines used in the study.

Name	Company	Catalog number
IL-6 ELISA kit	BD Biosciences	555220
IL-8 ELISA kit	BD Biosciences	555244
GM-CSF ELISA kit	BD Biosciences	555126
Mouse IL-6 ELISA kit	BD Biosciences	555240
Mouse CXCL1/KC ELISA kit	R&D Systems	DY453-05
Mouse CXCL2/MIP-2 ELISA kit	R&D Systems	DY452-05
