# Supplementary figures and images for: Aspartyl proteases target host actin nucleator complex protein to limit epithelial innate immunity
Source: EMBO Rep. 2024 Sep 30;25(11):4846–75. doi: 10.1038/s44319-024-00270-y (PMC11549443; doi:10.1038/s44319-024-00270-y)

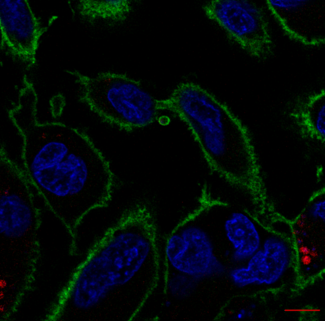

Supplement: Supplementary file 5 — Source data Fig. 1 [file 44319_2024_270_MOESM5_ESM.zip › Fig 1_Source data/Fig. 1C/Confocal images/Cgyps1-11del_24 h.tif]

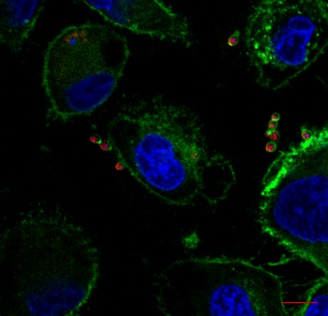

Supplement: Supplementary file 5 — Source data Fig. 1 [file 44319_2024_270_MOESM5_ESM.zip › Fig 1_Source data/Fig. 1C/Confocal images/Cgyps1-11del_4 h.tif]

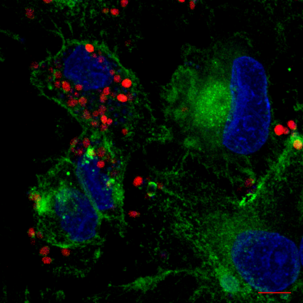

Supplement: Supplementary file 5 — Source data Fig. 1 [file 44319_2024_270_MOESM5_ESM.zip › Fig 1_Source data/Fig. 1C/Confocal images/wt_24 h.tif]

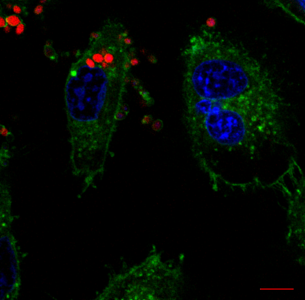

Supplement: Supplementary file 5 — Source data Fig. 1 [file 44319_2024_270_MOESM5_ESM.zip › Fig 1_Source data/Fig. 1C/Confocal images/wt_4 h.tif]

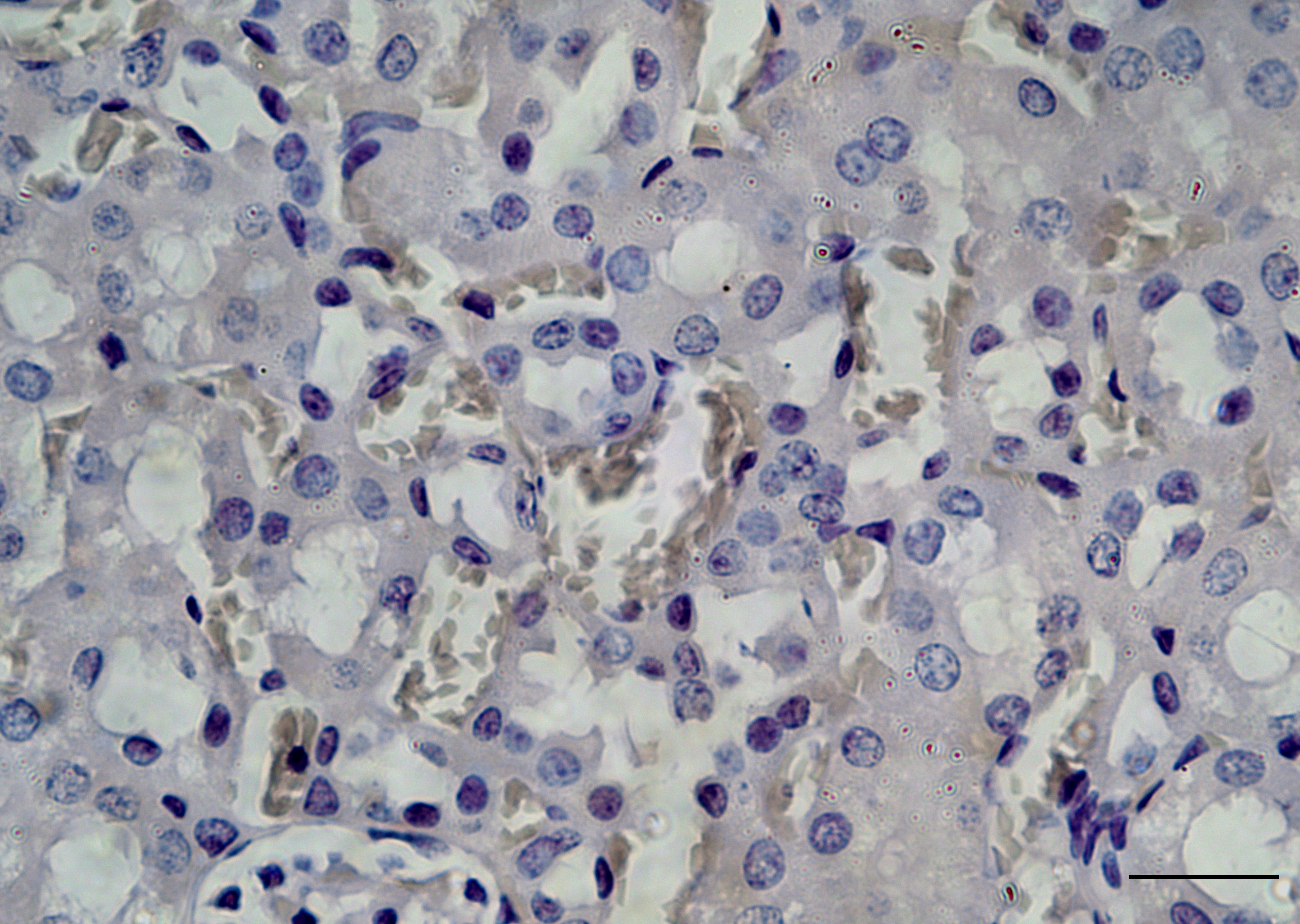

Supplement: Supplementary file 7 — Source data Fig. 3 [file 44319_2024_270_MOESM7_ESM.zip › Fig 3_Source data/Fig. 3D/1-11DEL Ly6G.tif]

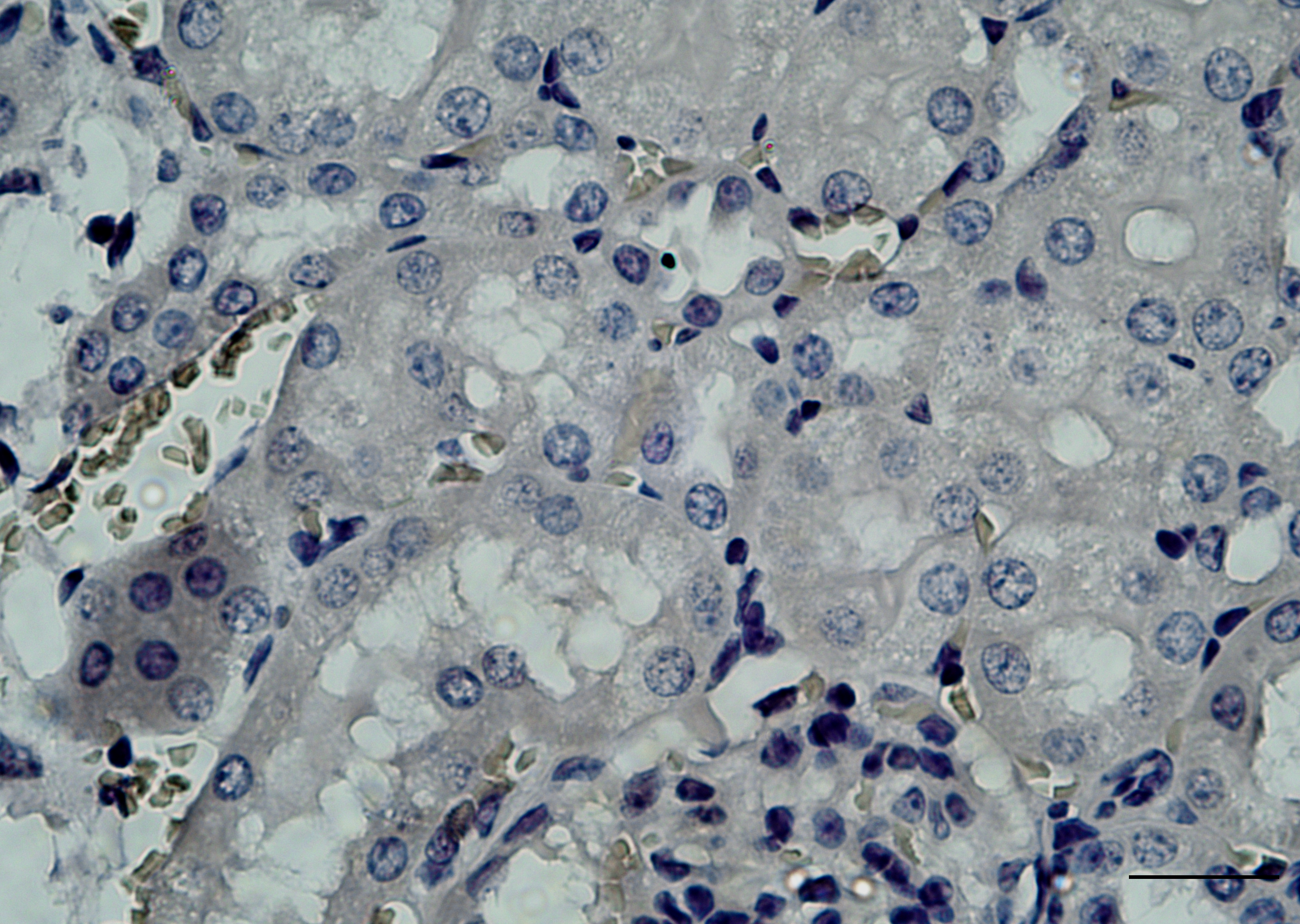

Supplement: Supplementary file 7 — Source data Fig. 3 [file 44319_2024_270_MOESM7_ESM.zip › Fig 3_Source data/Fig. 3D/Uninfected Ly6G.tif]

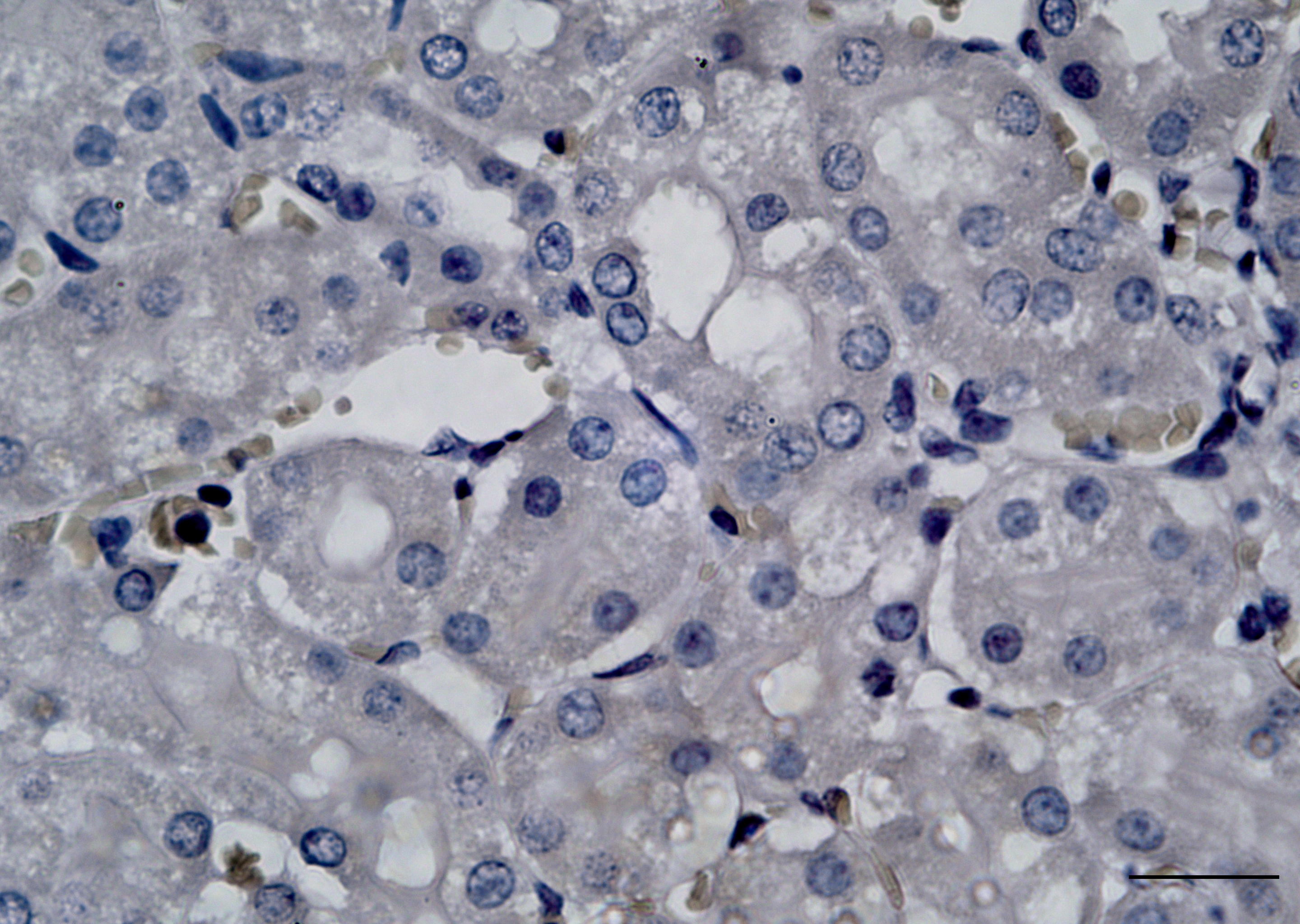

Supplement: Supplementary file 7 — Source data Fig. 3 [file 44319_2024_270_MOESM7_ESM.zip › Fig 3_Source data/Fig. 3D/wt Ly6G.tif]

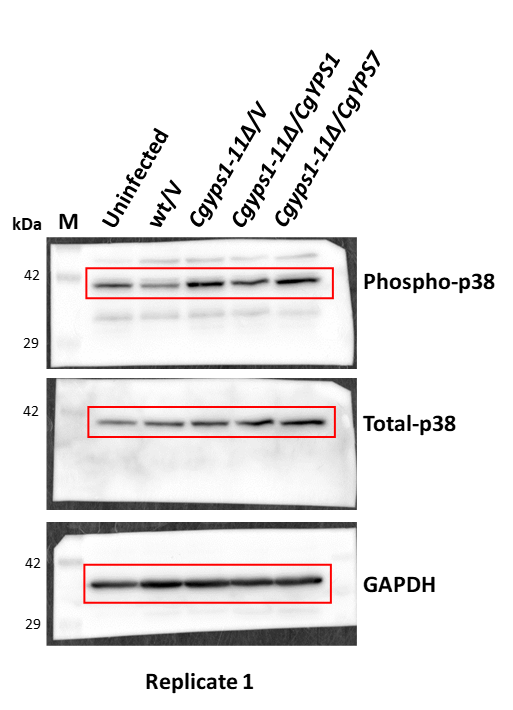

Supplement: Supplementary file 8 — Source data Fig. 4 [file 44319_2024_270_MOESM8_ESM.zip › Fig 4_Source data/Fig. 4A/Western blot_Phospho-p38.tif]

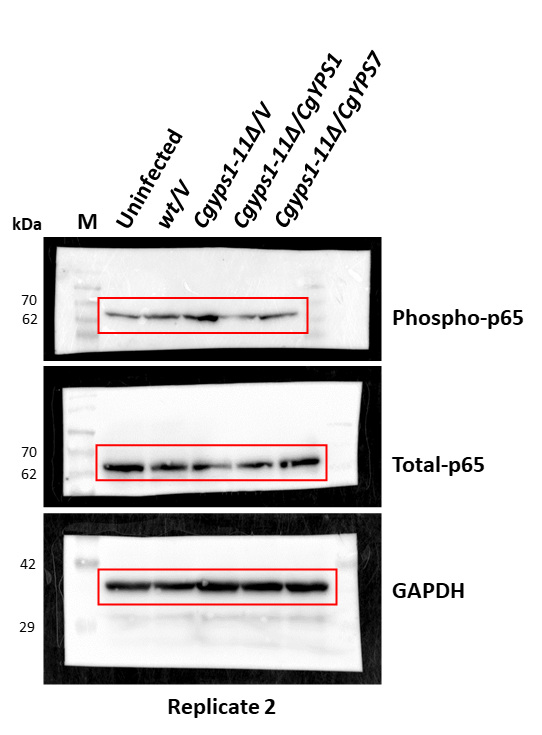

Supplement: Supplementary file 8 — Source data Fig. 4 [file 44319_2024_270_MOESM8_ESM.zip › Fig 4_Source data/Fig. 4B/Western blot_Phospho-p65.tif]

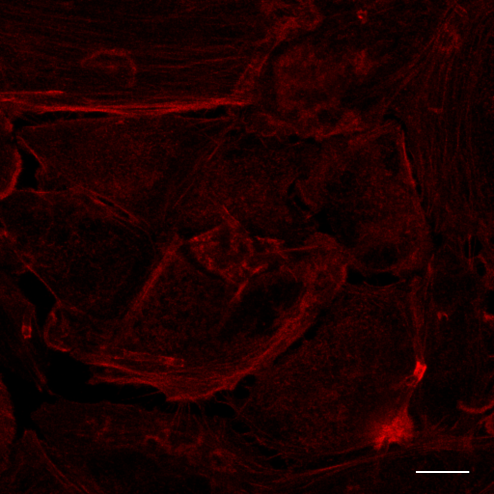

Supplement: Supplementary file 9 — Source data Fig. 5 [file 44319_2024_270_MOESM9_ESM.zip › Fig 5_Source data/Fig. 5C/C albicans A-498 ACTIN RHOD-PHALLOIDIN001_Series005Snapshot1_RAW_ch00.tif]

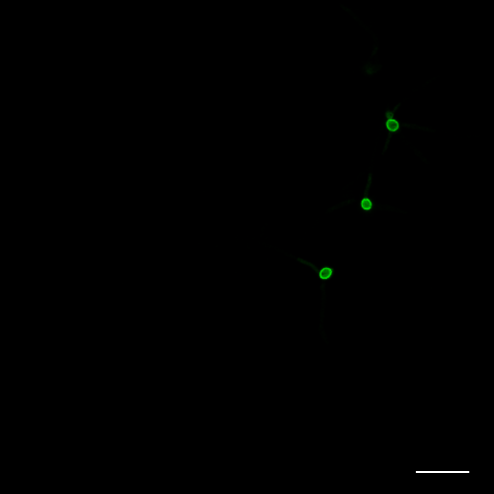

Supplement: Supplementary file 9 — Source data Fig. 5 [file 44319_2024_270_MOESM9_ESM.zip › Fig 5_Source data/Fig. 5C/C albicans A-498 ACTIN RHOD-PHALLOIDIN001_Series005Snapshot2_RAW_ch00.tif]

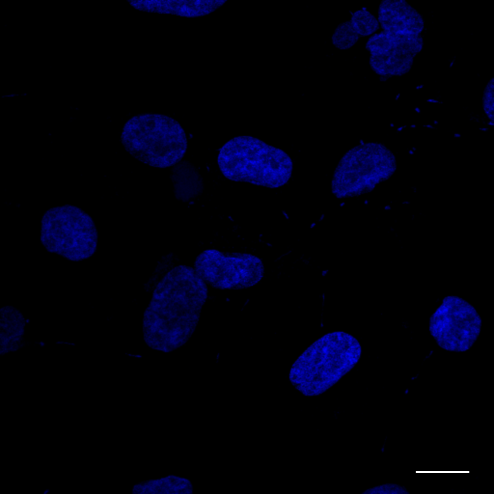

Supplement: Supplementary file 9 — Source data Fig. 5 [file 44319_2024_270_MOESM9_ESM.zip › Fig 5_Source data/Fig. 5C/C albicans A-498 ACTIN RHOD-PHALLOIDIN001_Series005Snapshot3_RAW_ch00.tif]

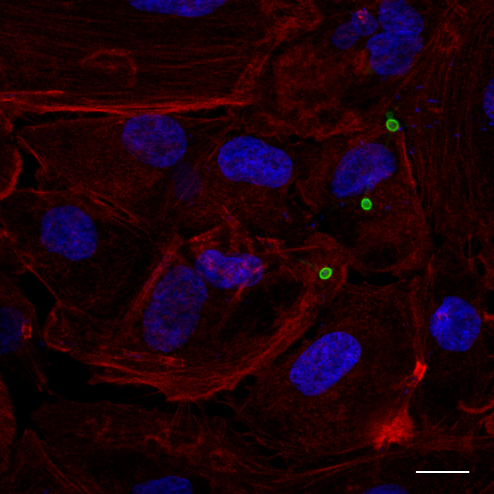

Supplement: Supplementary file 9 — Source data Fig. 5 [file 44319_2024_270_MOESM9_ESM.zip › Fig 5_Source data/Fig. 5C/C albicans A-498 ACTIN RHOD-PHALLOIDIN001_Series005Snapshot4_RAW_ch00.tif]

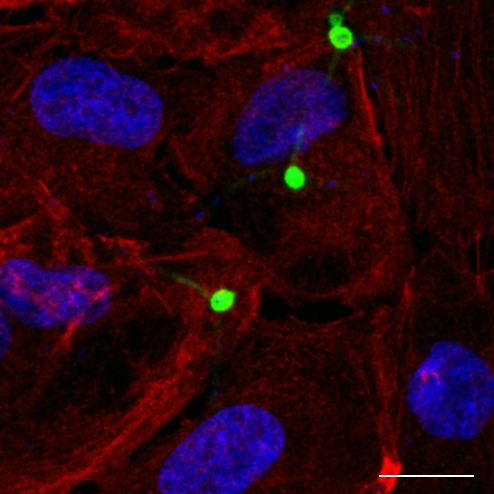

Supplement: Supplementary file 9 — Source data Fig. 5 [file 44319_2024_270_MOESM9_ESM.zip › Fig 5_Source data/Fig. 5C/C albicans A-498 ACTIN RHOD-PHALLOIDIN001_Series005_Crop002Snapshot1_RAW_ch00.tif]

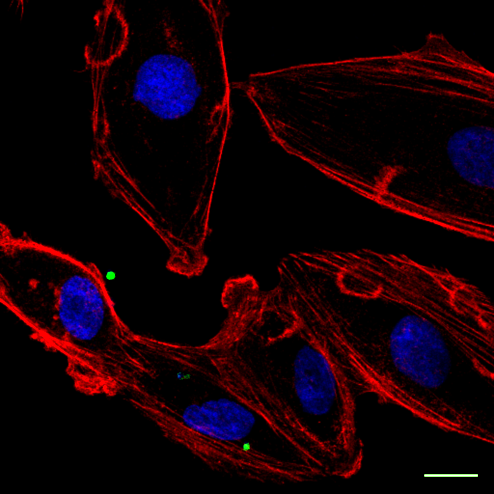

Supplement: Supplementary file 9 — Source data Fig. 5 [file 44319_2024_270_MOESM9_ESM.zip › Fig 5_Source data/Fig. 5C/Cgyps1-11 del A-498 ACTIN REPLICATE 2001_Series014Snapshot1 (2).tif]

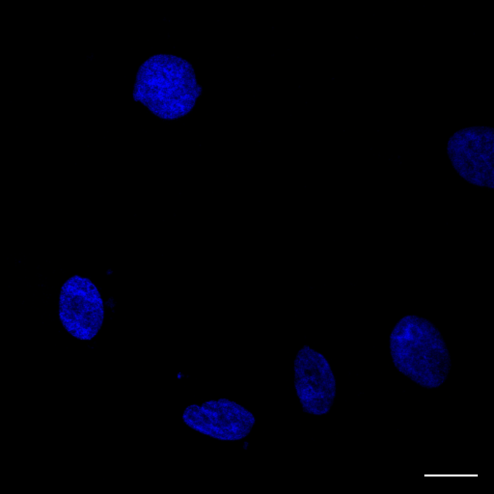

Supplement: Supplementary file 9 — Source data Fig. 5 [file 44319_2024_270_MOESM9_ESM.zip › Fig 5_Source data/Fig. 5C/Cgyps1-11 del A-498 ACTIN REPLICATE 2001_Series014Snapshot3_RAW_ch00.tif]

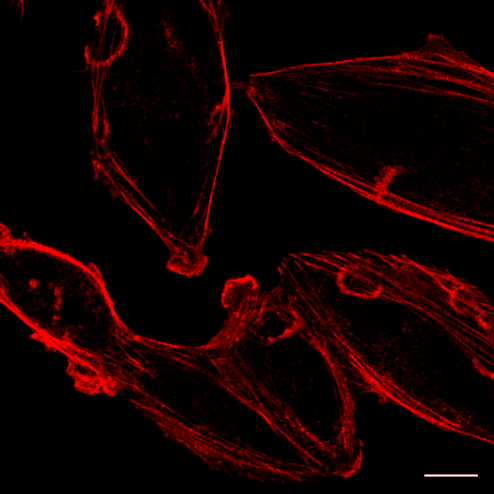

Supplement: Supplementary file 9 — Source data Fig. 5 [file 44319_2024_270_MOESM9_ESM.zip › Fig 5_Source data/Fig. 5C/Cgyps1-11 del A-498 ACTIN REPLICATE 2001_Series014Snapshot4.tif]

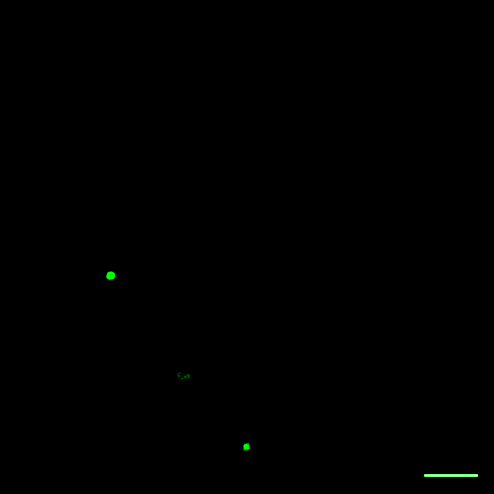

Supplement: Supplementary file 9 — Source data Fig. 5 [file 44319_2024_270_MOESM9_ESM.zip › Fig 5_Source data/Fig. 5C/Cgyps1-11 del A-498 ACTIN REPLICATE 2001_Series014Snapshot5.tif]

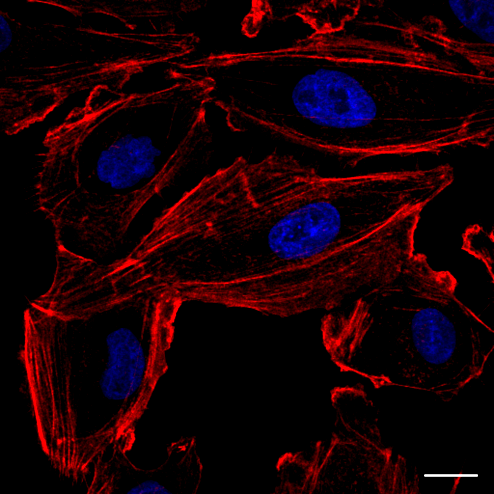

Supplement: Supplementary file 9 — Source data Fig. 5 [file 44319_2024_270_MOESM9_ESM.zip › Fig 5_Source data/Fig. 5C/Uninfected A-498 REPLICATE 2001_Series005Snapshot1.tif]

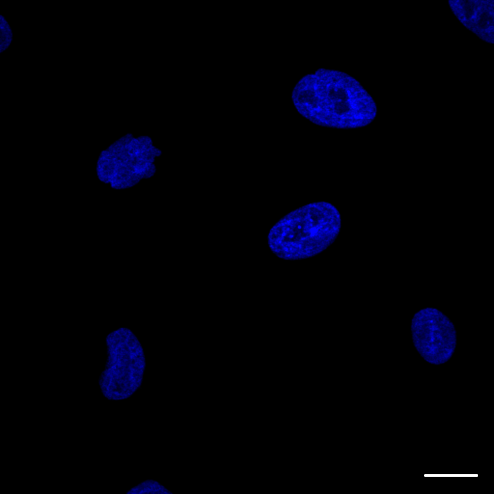

Supplement: Supplementary file 9 — Source data Fig. 5 [file 44319_2024_270_MOESM9_ESM.zip › Fig 5_Source data/Fig. 5C/Uninfected A-498 REPLICATE 2001_Series005Snapshot3.tif]

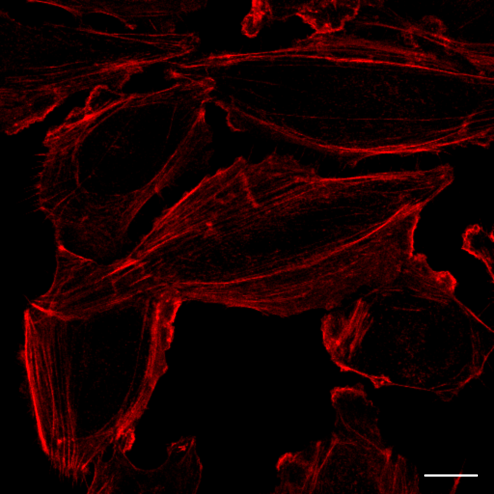

Supplement: Supplementary file 9 — Source data Fig. 5 [file 44319_2024_270_MOESM9_ESM.zip › Fig 5_Source data/Fig. 5C/Uninfected A-498 REPLICATE 2001_Series005Snapshot4.tif]

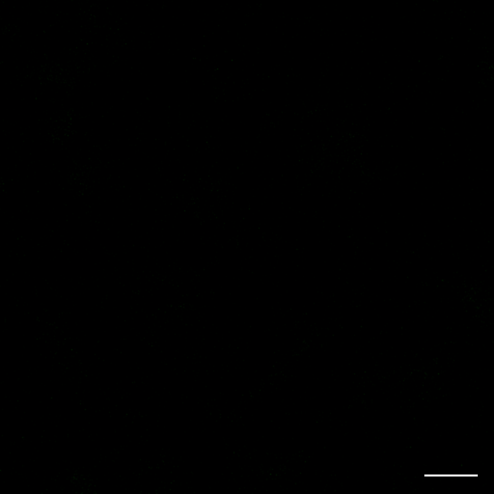

Supplement: Supplementary file 9 — Source data Fig. 5 [file 44319_2024_270_MOESM9_ESM.zip › Fig 5_Source data/Fig. 5C/Uninfected A-498 REPLICATE 2001_Series005Snapshot5_RAW_ch00.tif]

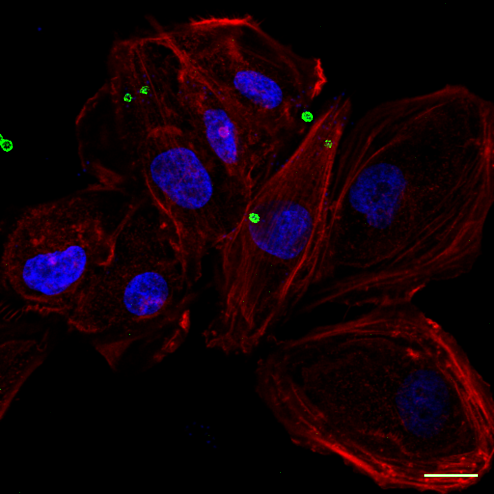

Supplement: Supplementary file 9 — Source data Fig. 5 [file 44319_2024_270_MOESM9_ESM.zip › Fig 5_Source data/Fig. 5C/WT A-498 ACTIN REPLICATE 2001_Series014Snapshot2.tif]

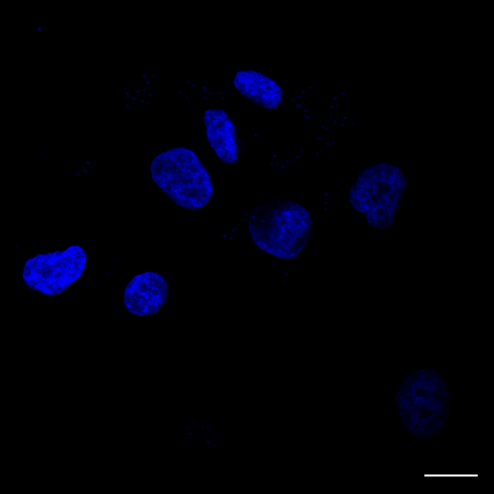

Supplement: Supplementary file 9 — Source data Fig. 5 [file 44319_2024_270_MOESM9_ESM.zip › Fig 5_Source data/Fig. 5C/WT A-498 ACTIN REPLICATE 2001_Series014Snapshot3_RAW_ch00.tif]

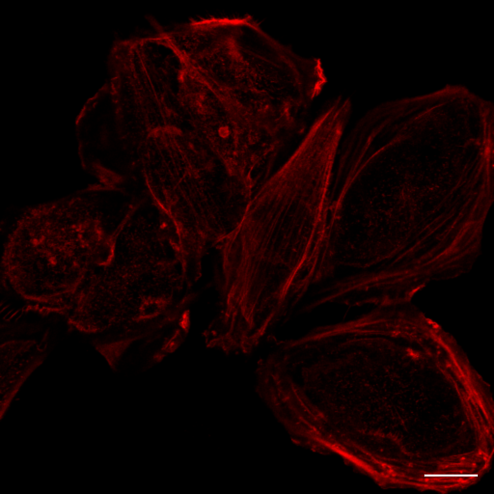

Supplement: Supplementary file 9 — Source data Fig. 5 [file 44319_2024_270_MOESM9_ESM.zip › Fig 5_Source data/Fig. 5C/WT A-498 ACTIN REPLICATE 2001_Series014Snapshot4_RAW_ch00.tif]

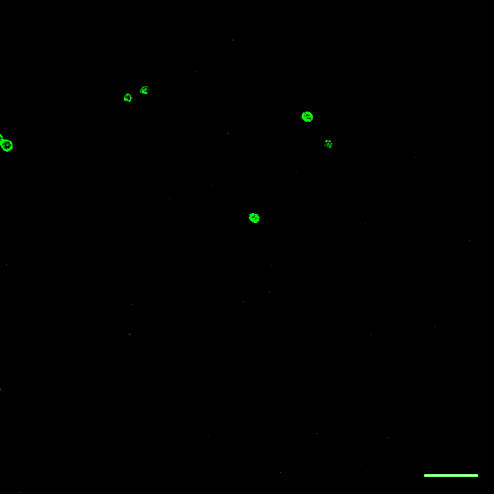

Supplement: Supplementary file 9 — Source data Fig. 5 [file 44319_2024_270_MOESM9_ESM.zip › Fig 5_Source data/Fig. 5C/WT A-498 ACTIN REPLICATE 2001_Series014Snapshot5.tif]

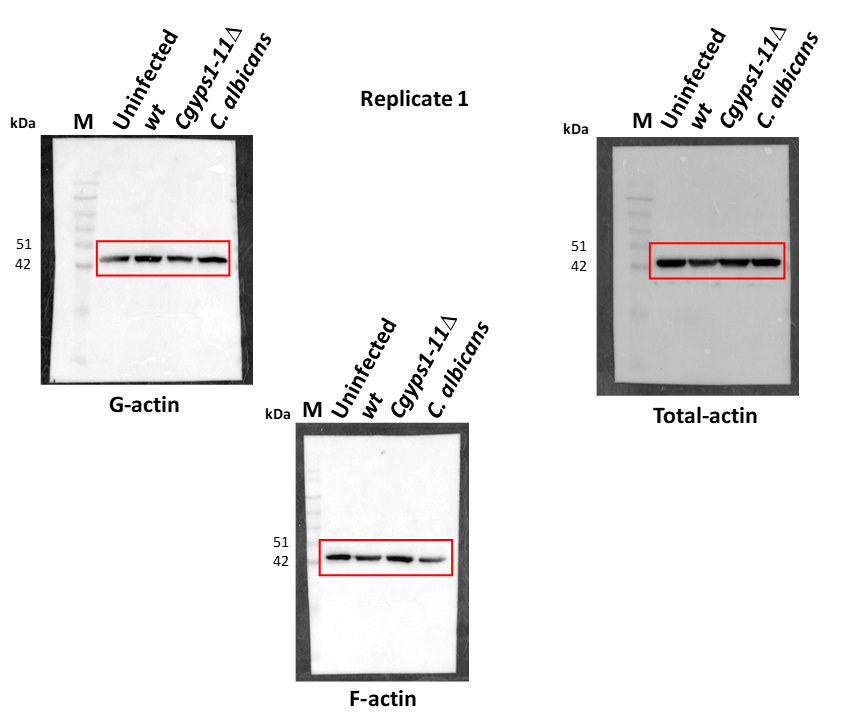

Supplement: Supplementary file 9 — Source data Fig. 5 [file 44319_2024_270_MOESM9_ESM.zip › Fig 5_Source data/Fig. 5D/Western blot_G-actin and F-actin.tif]

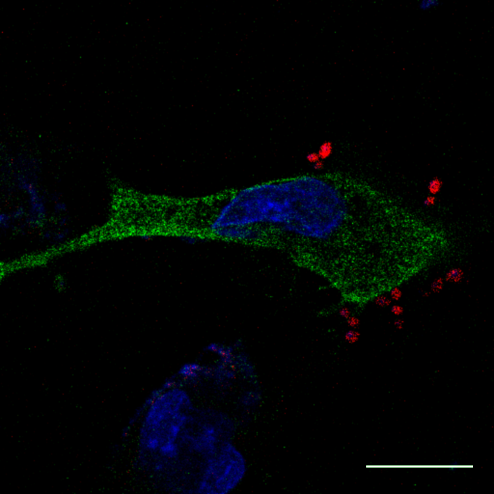

Supplement: Supplementary file 9 — Source data Fig. 5 [file 44319_2024_270_MOESM9_ESM.zip › Fig 5_Source data/Fig. 5E/1-11 del infected ARPC1B A-498 001_Series013Snapshot1.tif]

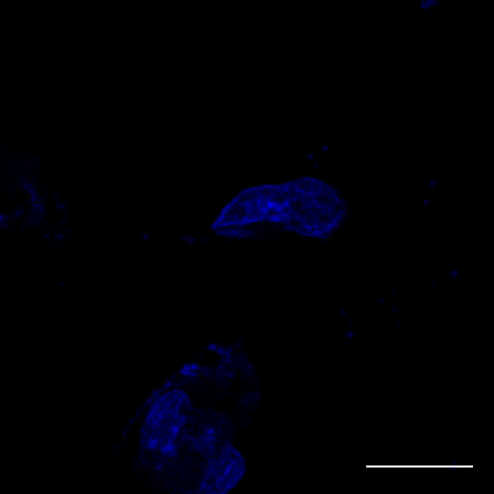

Supplement: Supplementary file 9 — Source data Fig. 5 [file 44319_2024_270_MOESM9_ESM.zip › Fig 5_Source data/Fig. 5E/1-11 del infected ARPC1B A-498 001_Series013Snapshot2_RAW_ch00.tif]

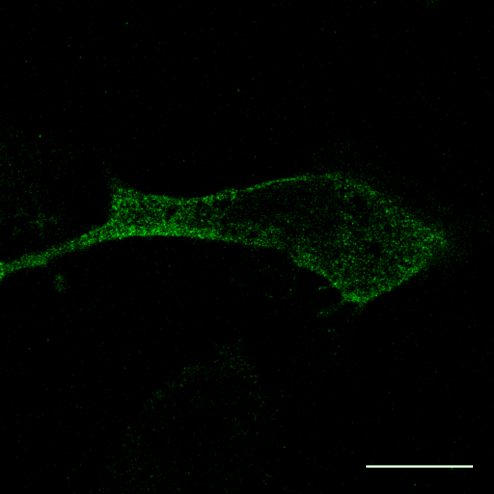

Supplement: Supplementary file 9 — Source data Fig. 5 [file 44319_2024_270_MOESM9_ESM.zip › Fig 5_Source data/Fig. 5E/1-11 del infected ARPC1B A-498 001_Series013Snapshot3.tif]

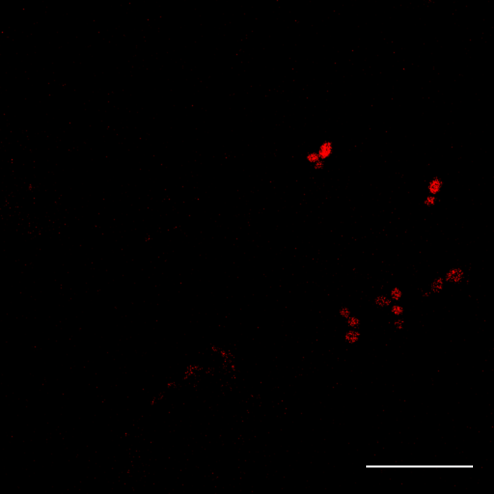

Supplement: Supplementary file 9 — Source data Fig. 5 [file 44319_2024_270_MOESM9_ESM.zip › Fig 5_Source data/Fig. 5E/1-11 del infected ARPC1B A-498 001_Series013Snapshot4_RAW_ch00.tif]

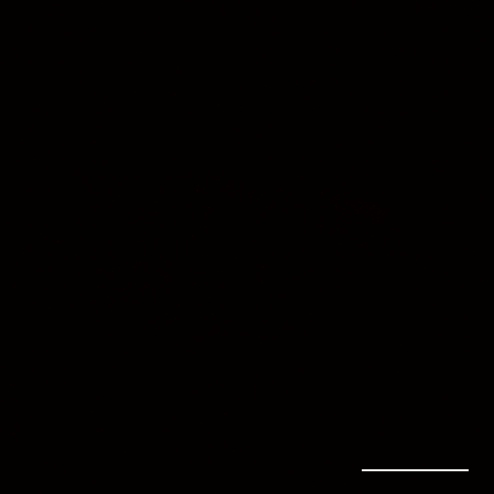

Supplement: Supplementary file 9 — Source data Fig. 5 [file 44319_2024_270_MOESM9_ESM.zip › Fig 5_Source data/Fig. 5E/Uninfected ARPC1B A-498 001_Series010Snapshot1_RAW_ch00.tif]

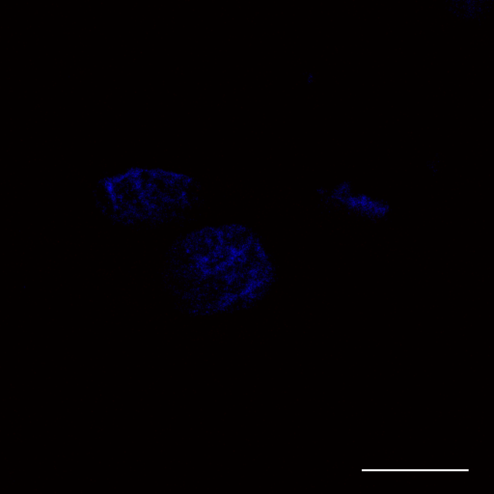

Supplement: Supplementary file 9 — Source data Fig. 5 [file 44319_2024_270_MOESM9_ESM.zip › Fig 5_Source data/Fig. 5E/Uninfected ARPC1B A-498 001_Series010Snapshot2_RAW_ch00.tif]

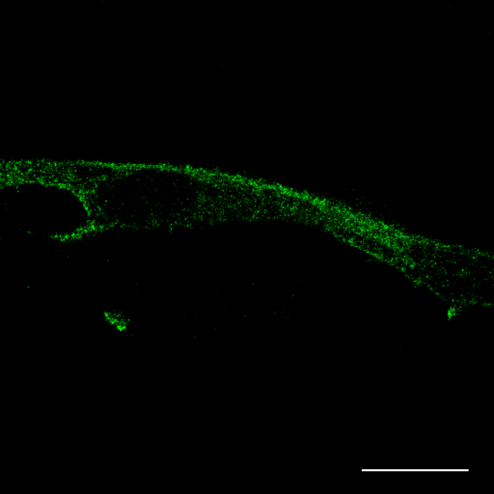

Supplement: Supplementary file 9 — Source data Fig. 5 [file 44319_2024_270_MOESM9_ESM.zip › Fig 5_Source data/Fig. 5E/Uninfected ARPC1B A-498 001_Series010Snapshot3_RAW_ch00.tif]

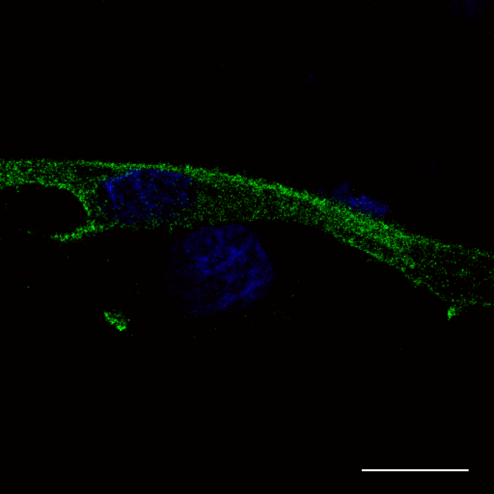

Supplement: Supplementary file 9 — Source data Fig. 5 [file 44319_2024_270_MOESM9_ESM.zip › Fig 5_Source data/Fig. 5E/Uninfected ARPC1B A-498 001_Series010Snapshot4_RAW_ch00.tif]

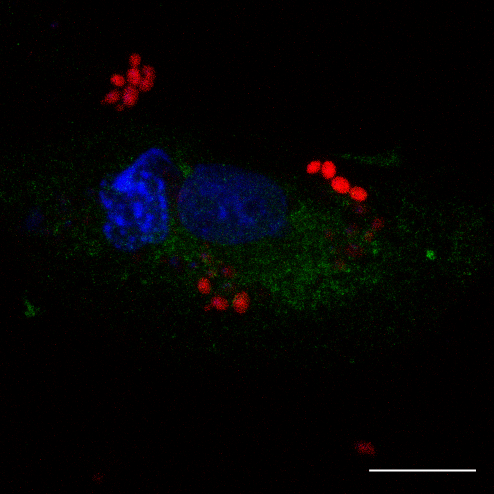

Supplement: Supplementary file 9 — Source data Fig. 5 [file 44319_2024_270_MOESM9_ESM.zip › Fig 5_Source data/Fig. 5E/WT ARPC1B A-498001_Series021Snapshot5_RAW_ch00 (2).tif]

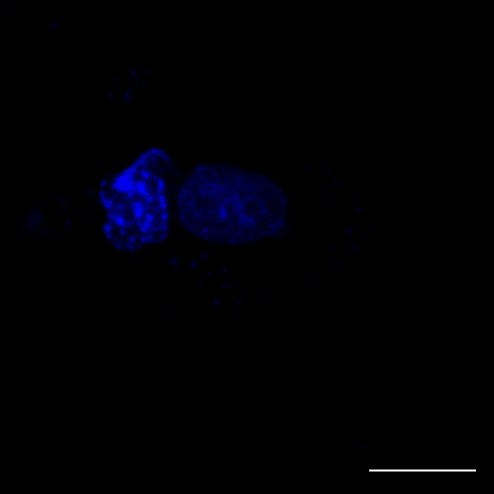

Supplement: Supplementary file 9 — Source data Fig. 5 [file 44319_2024_270_MOESM9_ESM.zip › Fig 5_Source data/Fig. 5E/WT ARPC1B A-498001_Series021Snapshot6_RAW_ch00 (2).tif]

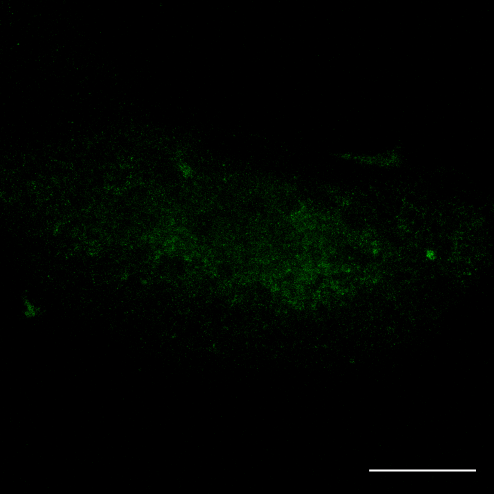

Supplement: Supplementary file 9 — Source data Fig. 5 [file 44319_2024_270_MOESM9_ESM.zip › Fig 5_Source data/Fig. 5E/WT ARPC1B A-498001_Series021Snapshot7_RAW_ch00 (2).tif]

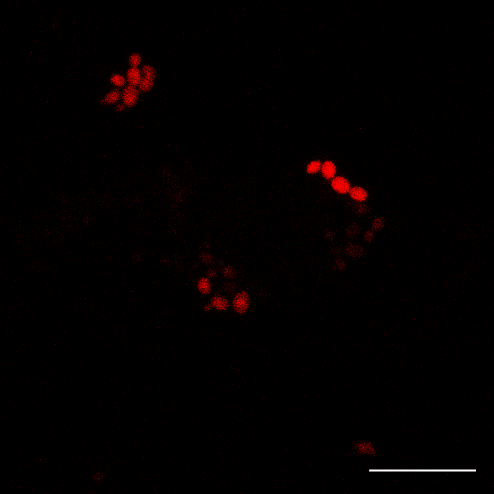

Supplement: Supplementary file 9 — Source data Fig. 5 [file 44319_2024_270_MOESM9_ESM.zip › Fig 5_Source data/Fig. 5E/WT ARPC1B A-498001_Series021Snapshot8_RAW_ch00 (2).tif]

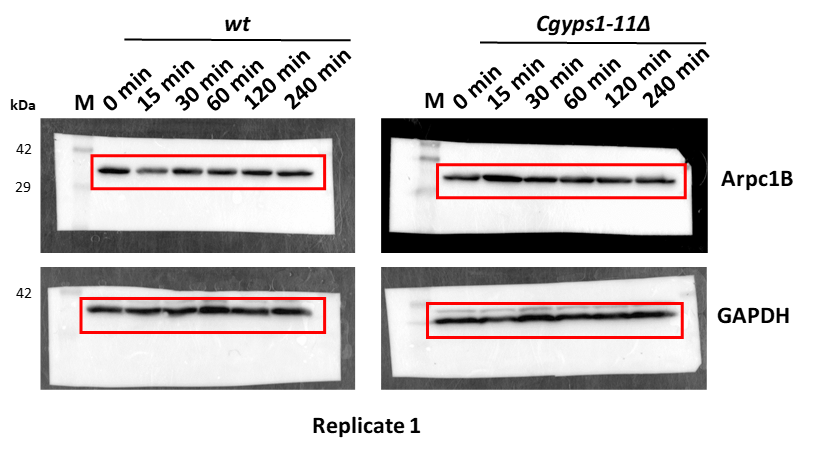

Supplement: Supplementary file 9 — Source data Fig. 5 [file 44319_2024_270_MOESM9_ESM.zip › Fig 5_Source data/Fig. 5F/Western blot_Arpc1B Kinetics.tif]

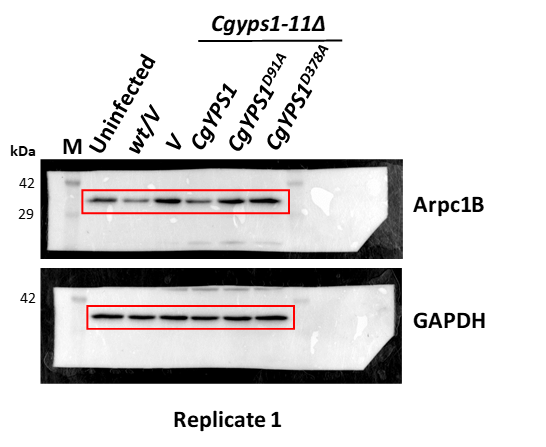

Supplement: Supplementary file 9 — Source data Fig. 5 [file 44319_2024_270_MOESM9_ESM.zip › Fig 5_Source data/Fig. 5G/Western blot_Arpc1B_CgYapsin complemented strains.tif]

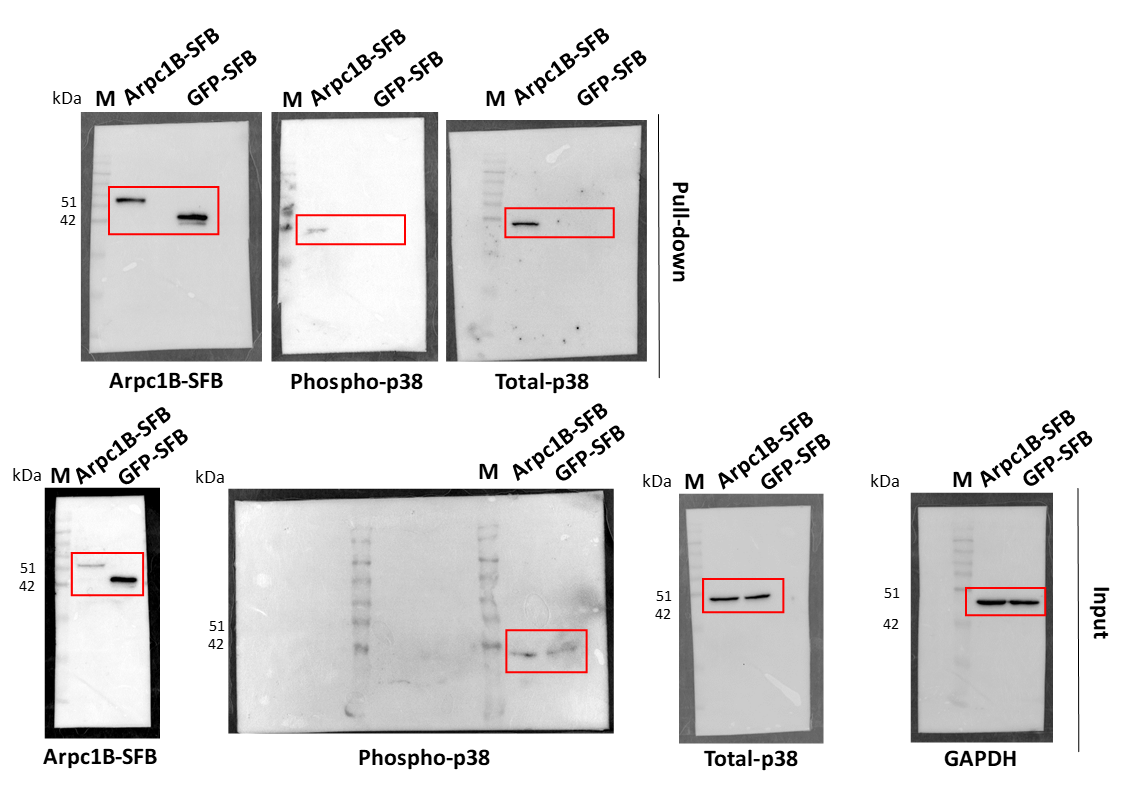

Supplement: Supplementary file 9 — Source data Fig. 5 [file 44319_2024_270_MOESM9_ESM.zip › Fig 5_Source data/Fig. 5H/Western blot_Arpc1B-p38 interaction.tif]

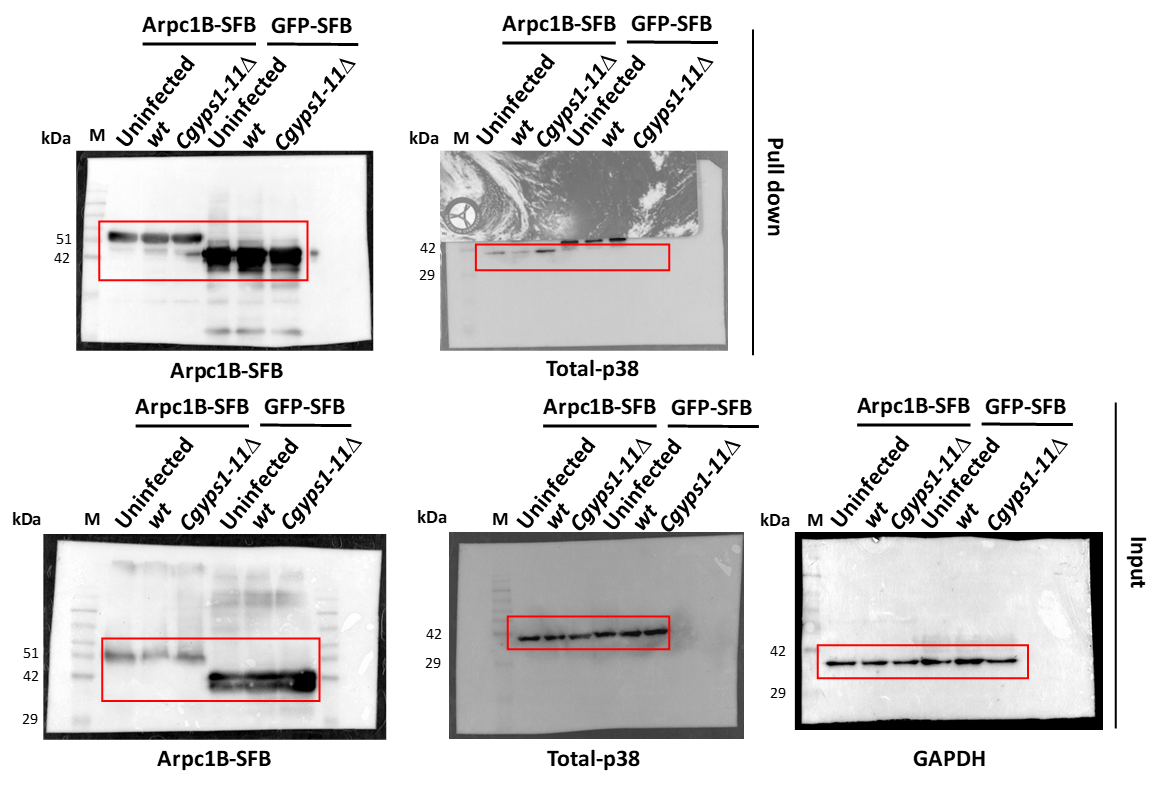

Supplement: Supplementary file 9 — Source data Fig. 5 [file 44319_2024_270_MOESM9_ESM.zip › Fig 5_Source data/Fig. 5I/Wetern blot_Cg-infected A-498 Aprc1B-p38 interaction_quantification.tif]

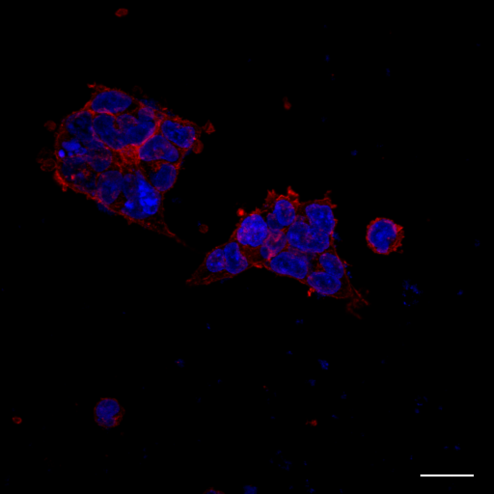

Supplement: Supplementary file 11 — Source data Fig. 7 [file 44319_2024_270_MOESM11_ESM.zip › Fig 7_Source data/Fig. 7A/Arpc1B KO_HEK 293T_Replicate 1001_Series001Snapshot2_RAW_ch00.tif]

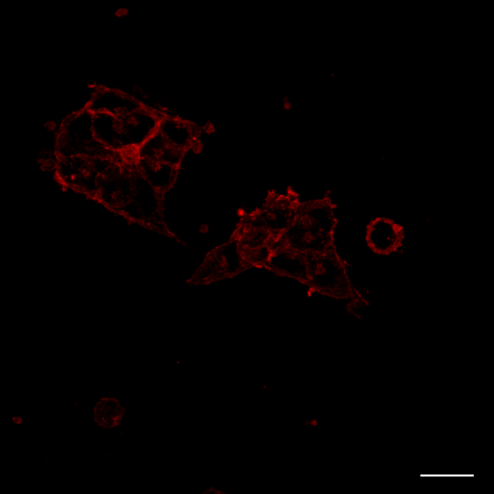

Supplement: Supplementary file 11 — Source data Fig. 7 [file 44319_2024_270_MOESM11_ESM.zip › Fig 7_Source data/Fig. 7A/Arpc1B KO_HEK 293T_Replicate 1001_Series001Snapshot3_RAW_ch00.tif]

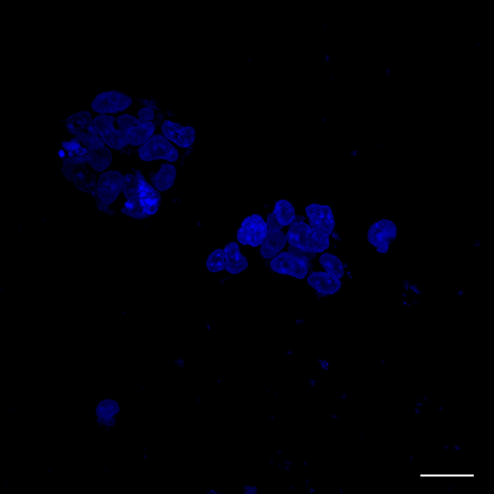

Supplement: Supplementary file 11 — Source data Fig. 7 [file 44319_2024_270_MOESM11_ESM.zip › Fig 7_Source data/Fig. 7A/Arpc1B KO_HEK 293T_Replicate 1001_Series001Snapshot4_RAW_ch00.tif]

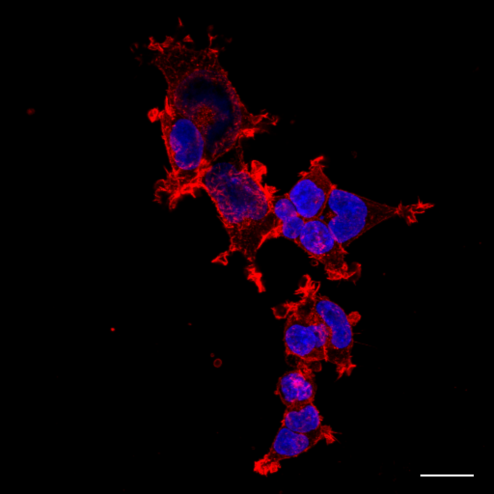

Supplement: Supplementary file 11 — Source data Fig. 7 [file 44319_2024_270_MOESM11_ESM.zip › Fig 7_Source data/Fig. 7A/WT_NTC_HEK 293T_Replicate 1001_Series009Snapshot2.tif]

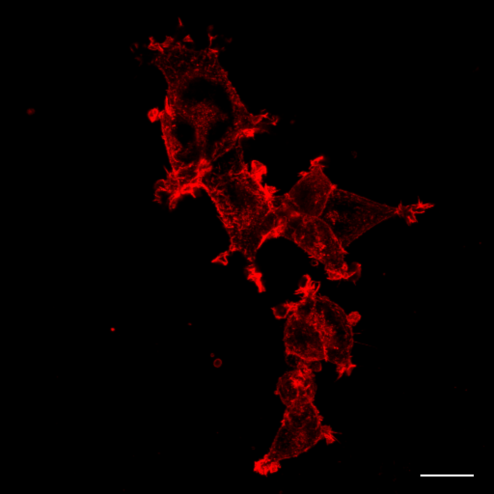

Supplement: Supplementary file 11 — Source data Fig. 7 [file 44319_2024_270_MOESM11_ESM.zip › Fig 7_Source data/Fig. 7A/WT_NTC_HEK 293T_Replicate 1001_Series009Snapshot3_RAW_ch00.tif]

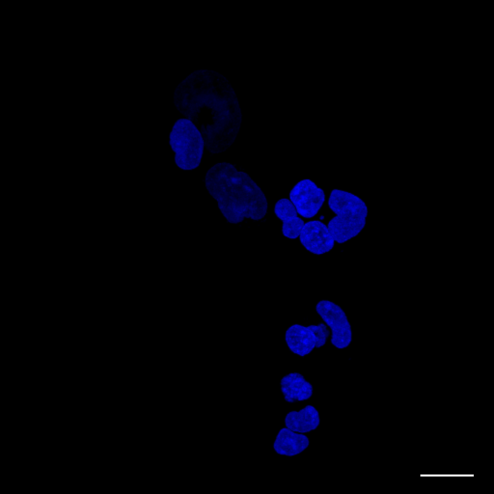

Supplement: Supplementary file 11 — Source data Fig. 7 [file 44319_2024_270_MOESM11_ESM.zip › Fig 7_Source data/Fig. 7A/WT_NTC_HEK 293T_Replicate 1001_Series009Snapshot4_RAW_ch00.tif]

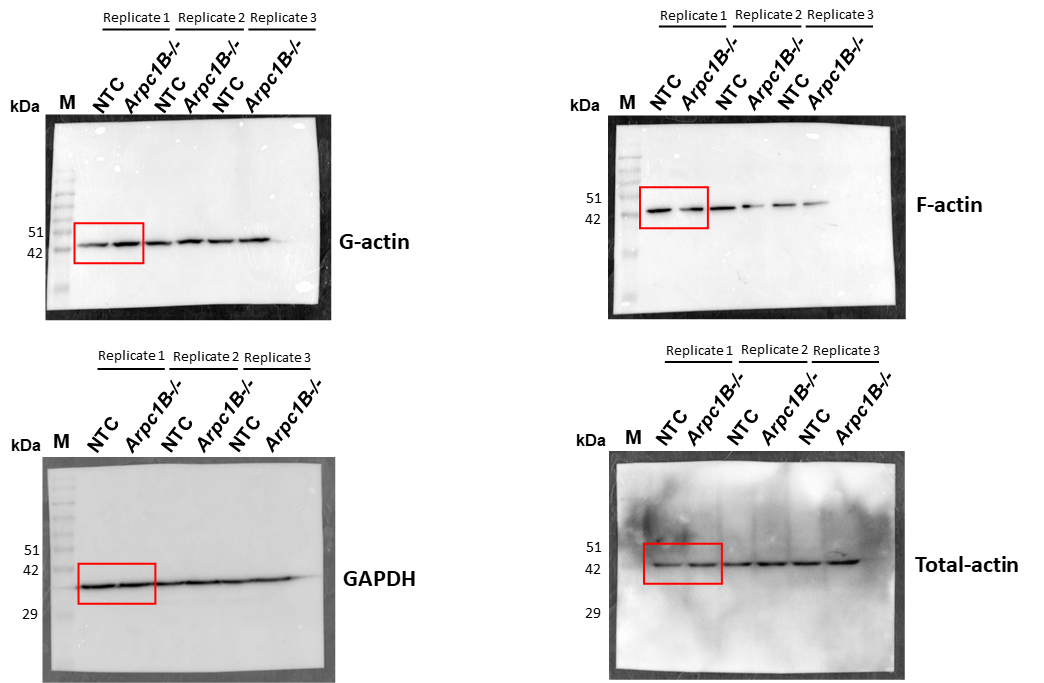

Supplement: Supplementary file 11 — Source data Fig. 7 [file 44319_2024_270_MOESM11_ESM.zip › Fig 7_Source data/Fig. 7B/Western blot_F-Actin and G-actin ratio.tif]

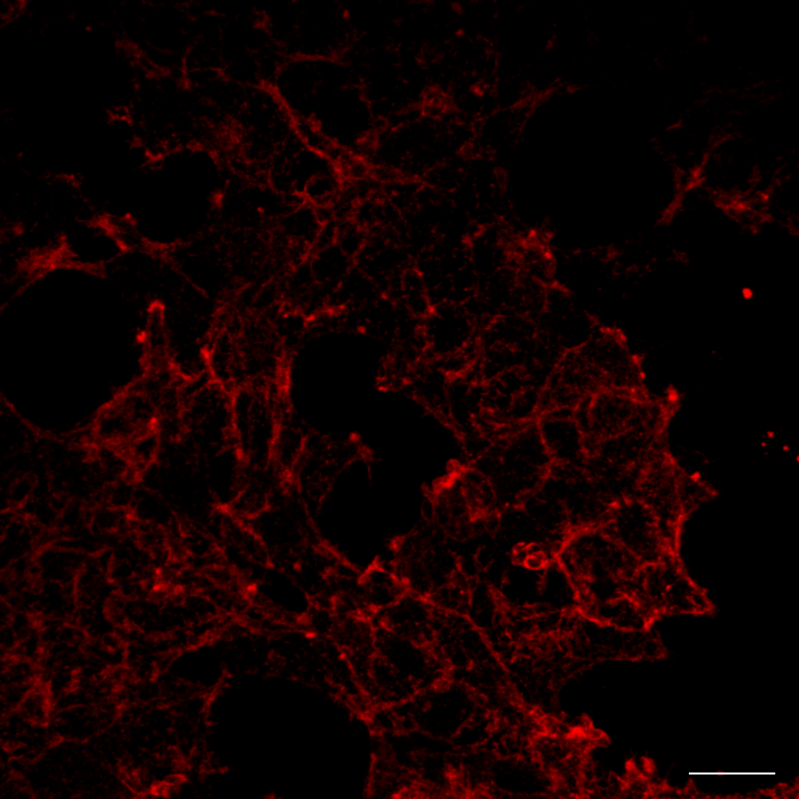

Supplement: Supplementary file 11 — Source data Fig. 7 [file 44319_2024_270_MOESM11_ESM.zip › Fig 7_Source data/Fig. 7C/Arpc1B KO/Series015Snapshot2_RAW_ch00.tif]

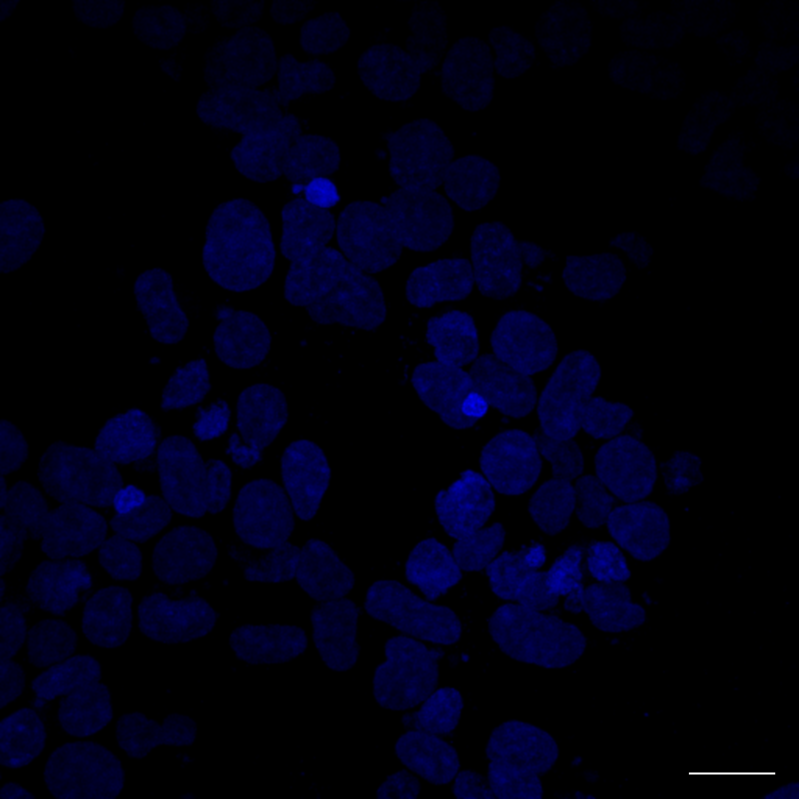

Supplement: Supplementary file 11 — Source data Fig. 7 [file 44319_2024_270_MOESM11_ESM.zip › Fig 7_Source data/Fig. 7C/Arpc1B KO/Series015Snapshot3_RAW_ch00.tif]

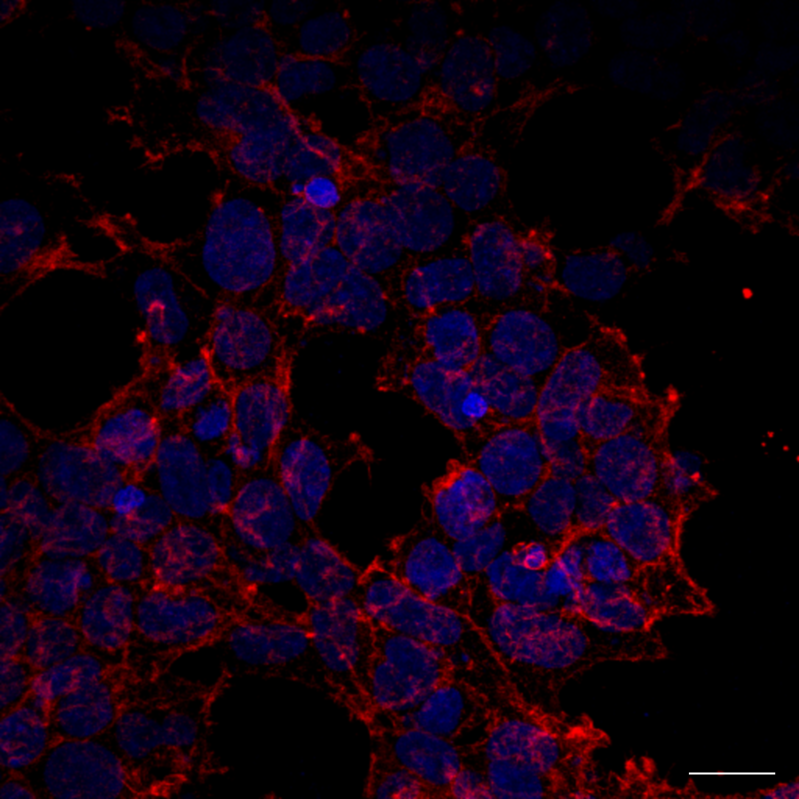

Supplement: Supplementary file 11 — Source data Fig. 7 [file 44319_2024_270_MOESM11_ESM.zip › Fig 7_Source data/Fig. 7C/Arpc1B KO/Series015Snapshot4_RAW_ch00.tif]

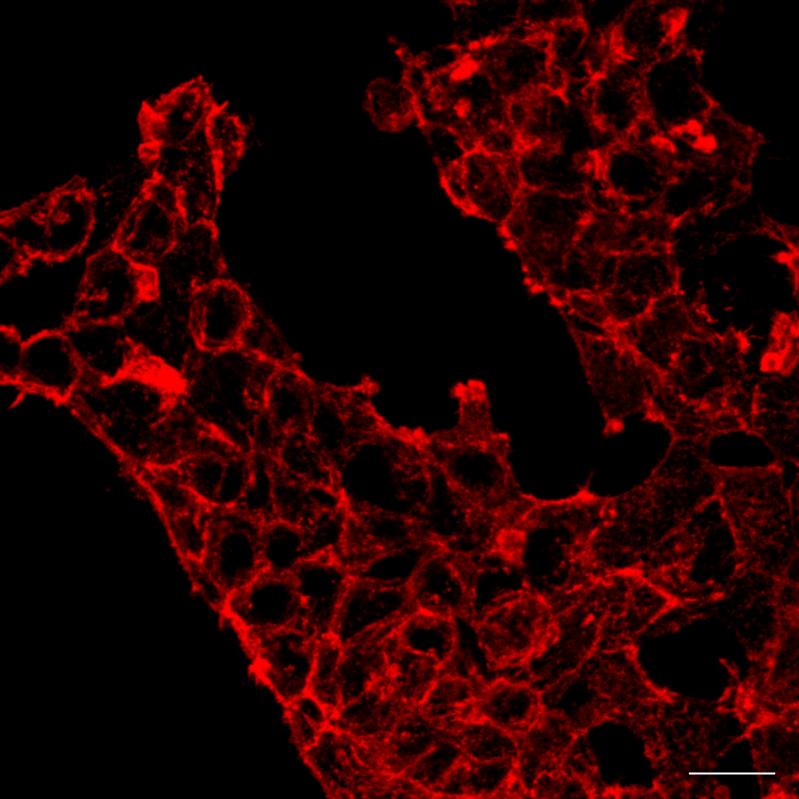

Supplement: Supplementary file 11 — Source data Fig. 7 [file 44319_2024_270_MOESM11_ESM.zip › Fig 7_Source data/Fig. 7C/Arpc1B KO + Arpc1B/Series014Snapshot2_RAW_ch00.tif]

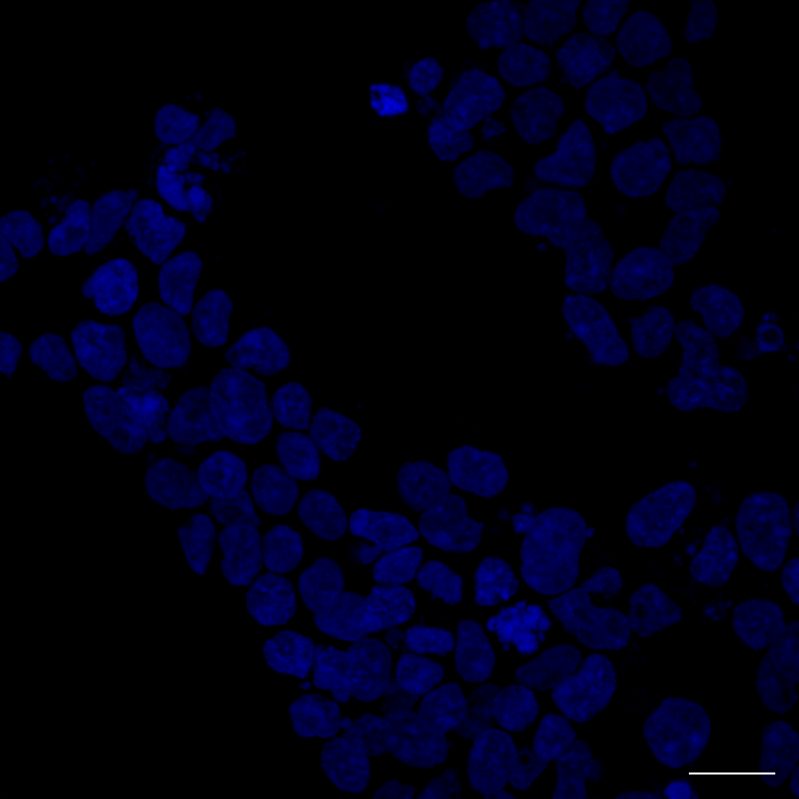

Supplement: Supplementary file 11 — Source data Fig. 7 [file 44319_2024_270_MOESM11_ESM.zip › Fig 7_Source data/Fig. 7C/Arpc1B KO + Arpc1B/Series014Snapshot3_RAW_ch00.tif]

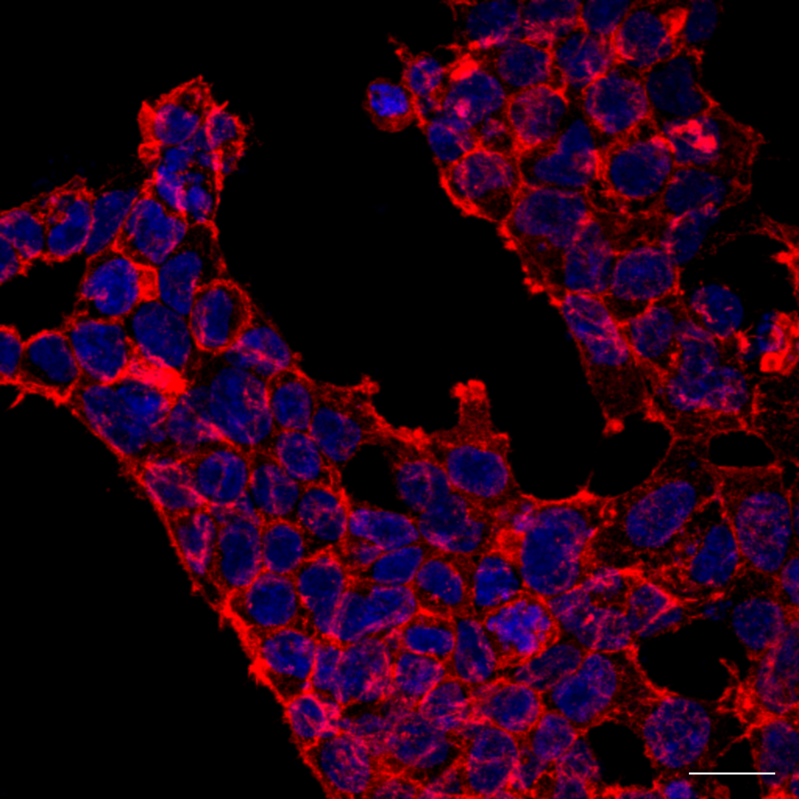

Supplement: Supplementary file 11 — Source data Fig. 7 [file 44319_2024_270_MOESM11_ESM.zip › Fig 7_Source data/Fig. 7C/Arpc1B KO + Arpc1B/Series014Snapshot4_RAW_ch00.tif]

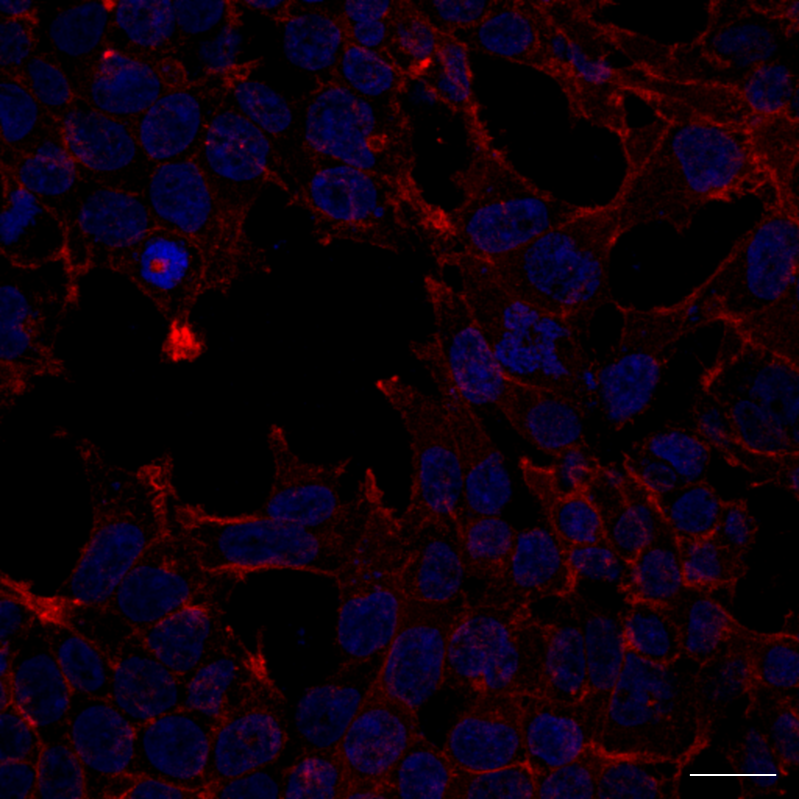

Supplement: Supplementary file 11 — Source data Fig. 7 [file 44319_2024_270_MOESM11_ESM.zip › Fig 7_Source data/Fig. 7C/Arpc1B SDM R142A/Series011Snapshot2_RAW_ch00.tif]

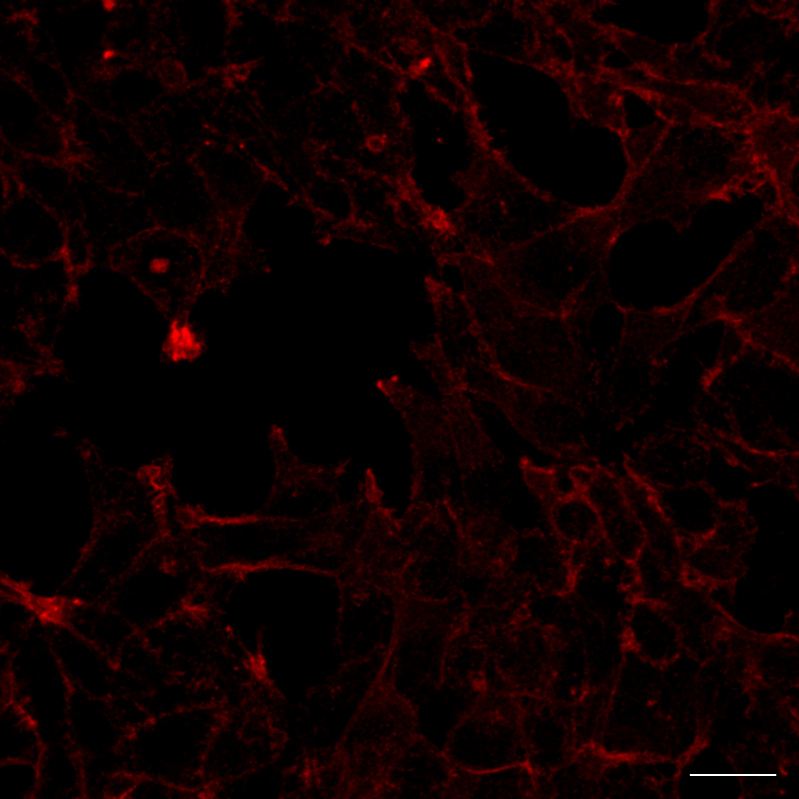

Supplement: Supplementary file 11 — Source data Fig. 7 [file 44319_2024_270_MOESM11_ESM.zip › Fig 7_Source data/Fig. 7C/Arpc1B SDM R142A/Series011Snapshot3_RAW_ch00.tif]

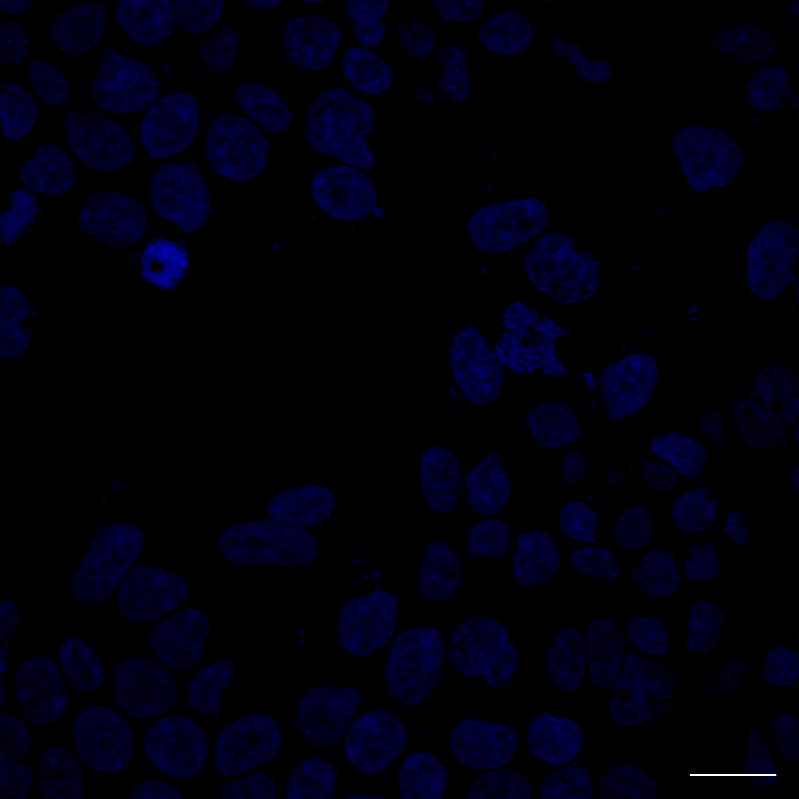

Supplement: Supplementary file 11 — Source data Fig. 7 [file 44319_2024_270_MOESM11_ESM.zip › Fig 7_Source data/Fig. 7C/Arpc1B SDM R142A/Series011Snapshot4_RAW_ch00.tif]

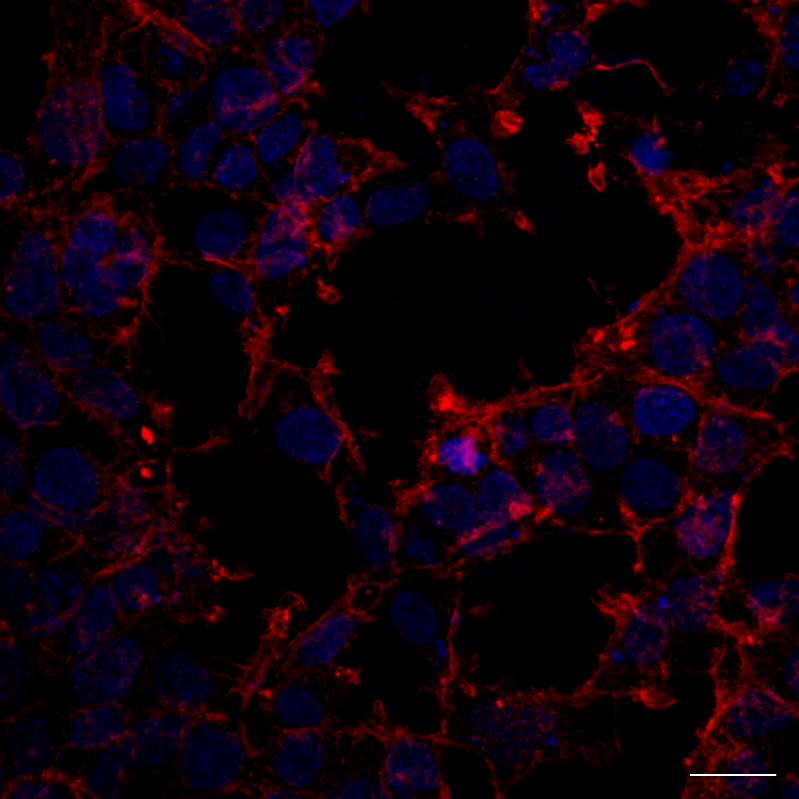

Supplement: Supplementary file 11 — Source data Fig. 7 [file 44319_2024_270_MOESM11_ESM.zip › Fig 7_Source data/Fig. 7C/Arpc1B SDM R74A/Series001Snapshot2.tif]

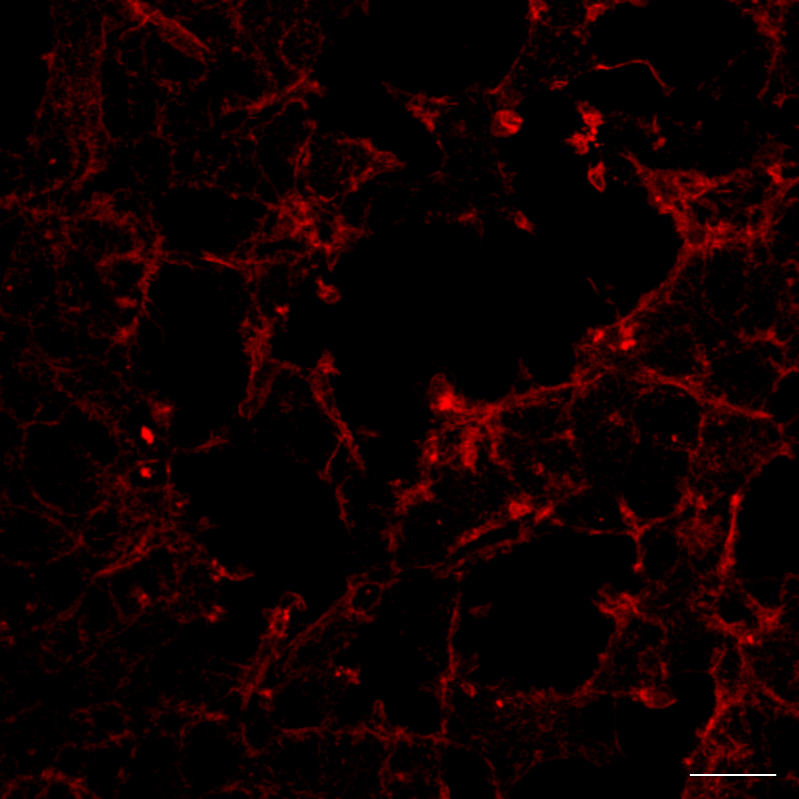

Supplement: Supplementary file 11 — Source data Fig. 7 [file 44319_2024_270_MOESM11_ESM.zip › Fig 7_Source data/Fig. 7C/Arpc1B SDM R74A/Series001Snapshot3_RAW_ch00.tif]

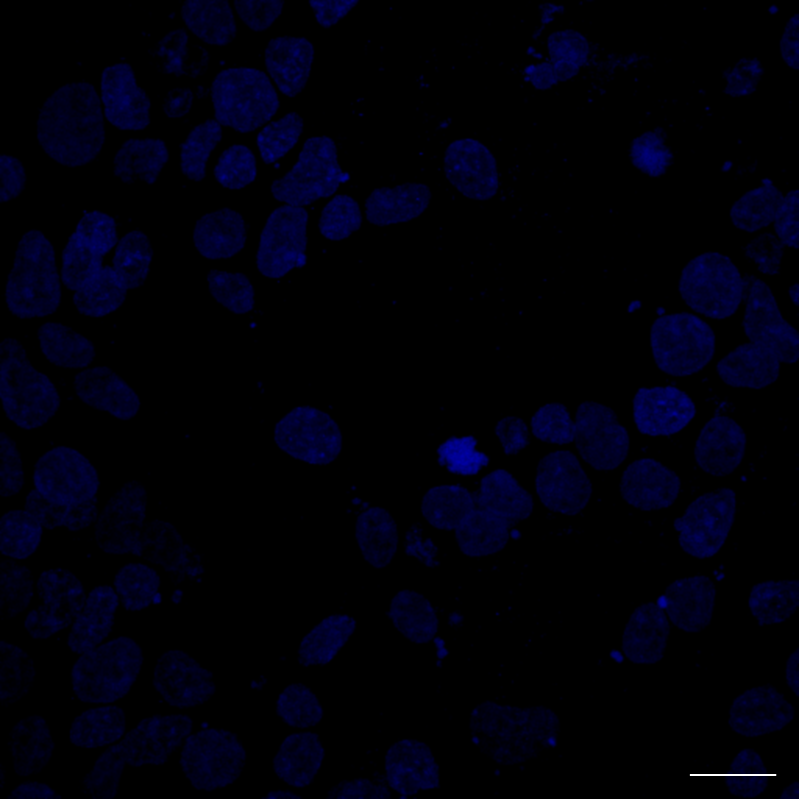

Supplement: Supplementary file 11 — Source data Fig. 7 [file 44319_2024_270_MOESM11_ESM.zip › Fig 7_Source data/Fig. 7C/Arpc1B SDM R74A/Series001Snapshot4_RAW_ch00.tif]

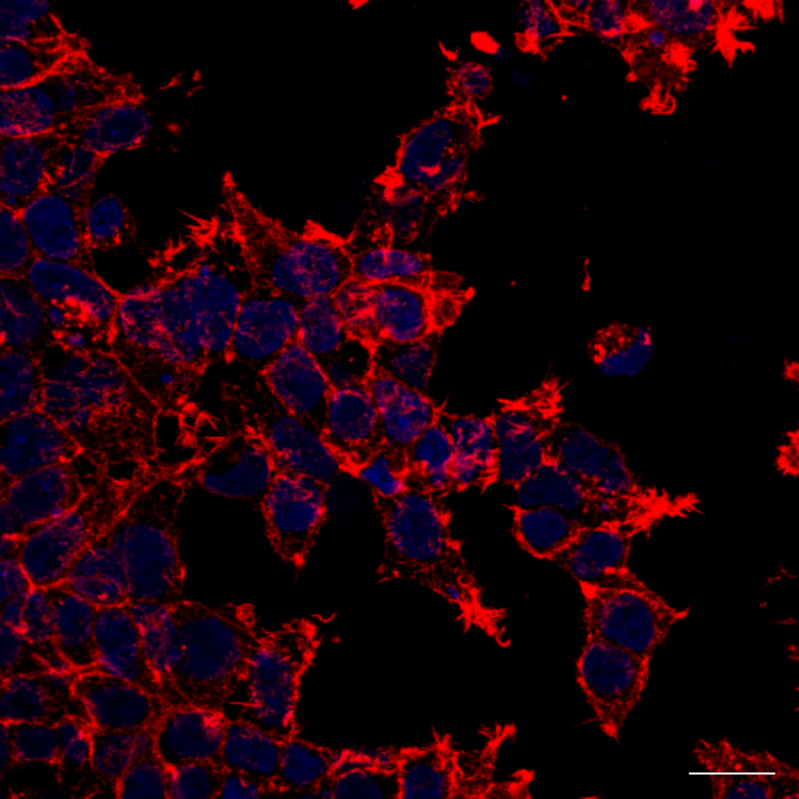

Supplement: Supplementary file 11 — Source data Fig. 7 [file 44319_2024_270_MOESM11_ESM.zip › Fig 7_Source data/Fig. 7C/NTC/Series007Snapshot2_RAW_ch00_NTC.tif]

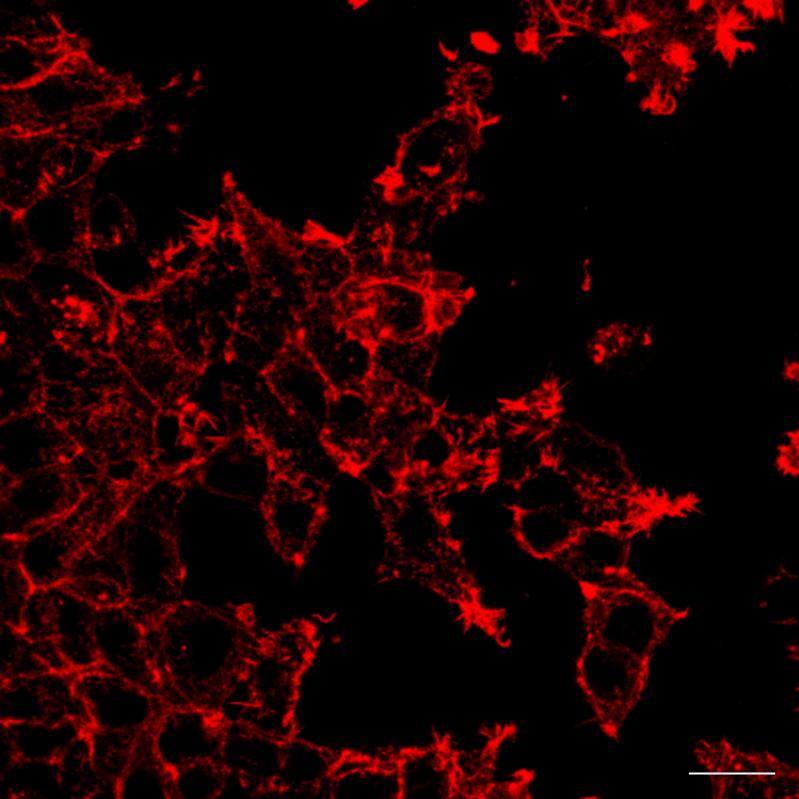

Supplement: Supplementary file 11 — Source data Fig. 7 [file 44319_2024_270_MOESM11_ESM.zip › Fig 7_Source data/Fig. 7C/NTC/Series007Snapshot3_RAW_ch00 NTC.tif]

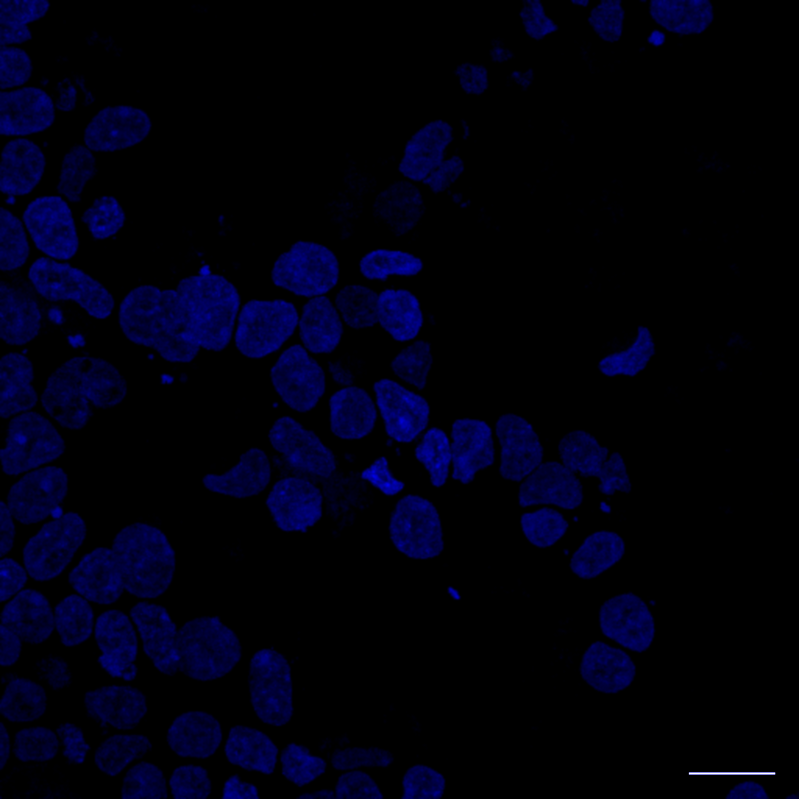

Supplement: Supplementary file 11 — Source data Fig. 7 [file 44319_2024_270_MOESM11_ESM.zip › Fig 7_Source data/Fig. 7C/NTC/Series007Snapshot4 DAPI NTC.tif]

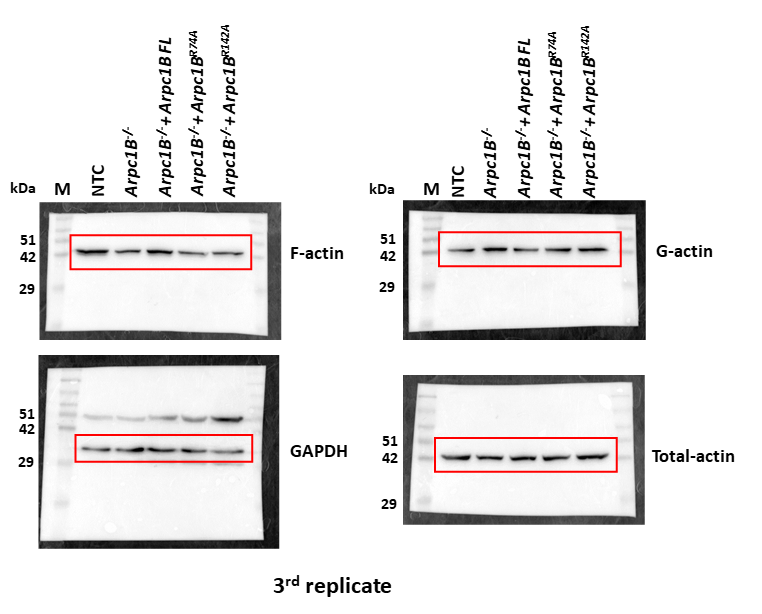

Supplement: Supplementary file 11 — Source data Fig. 7 [file 44319_2024_270_MOESM11_ESM.zip › Fig 7_Source data/Fig. 7D/Western blot_KO complementation f to g actin ratio.tif]

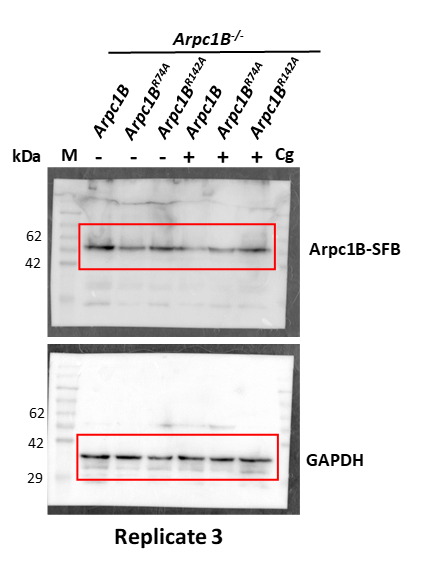

Supplement: Supplementary file 11 — Source data Fig. 7 [file 44319_2024_270_MOESM11_ESM.zip › Fig 7_Source data/Fig. 7E/Arpc1B SDM Quantification.tif]

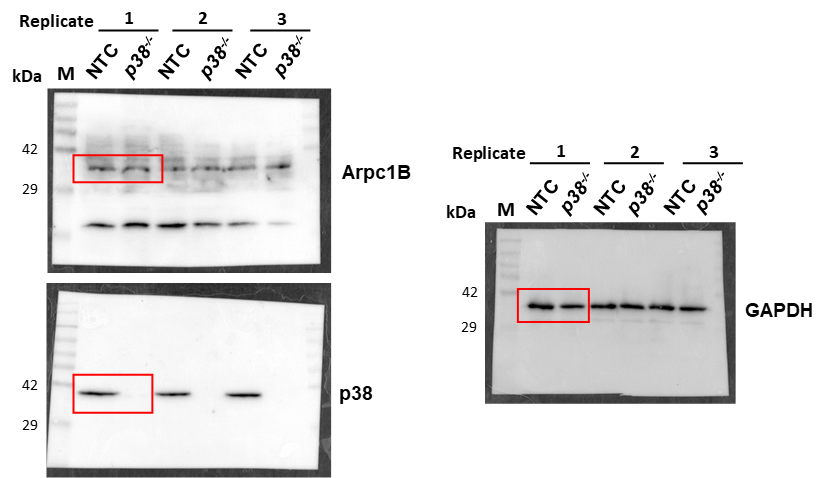

Supplement: Supplementary file 11 — Source data Fig. 7 [file 44319_2024_270_MOESM11_ESM.zip › Fig 7_Source data/Fig. 7H/Fig 7H_Western blot_Arpc1B_p38 KO.tif]

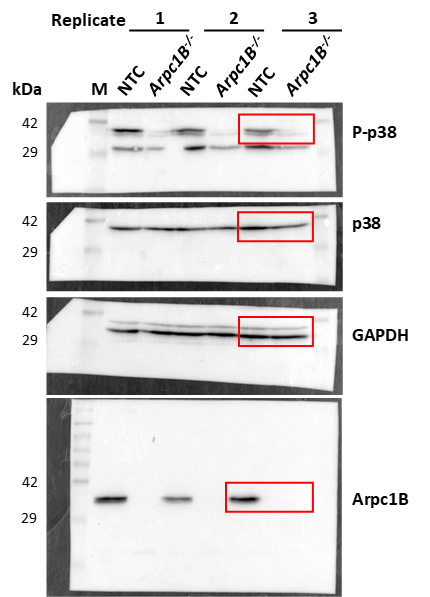

Supplement: Supplementary file 11 — Source data Fig. 7 [file 44319_2024_270_MOESM11_ESM.zip › Fig 7_Source data/Fig. 7I/Fig. 7I_Western blot_phospho p38_quantification_Arpc1B KO.tif]
