# Supplementary material for: Aspartyl proteases target host actin nucleator complex protein to limit epithelial innate immunity
Source: EMBO Rep. 2024 Sep 30;25(11):4846–75. doi: 10.1038/s44319-024-00270-y (PMC11549443; doi:10.1038/s44319-024-00270-y)
Supplement: Supplementary file 13 — Expanded View Figures [file 44319_2024_270_MOESM13_ESM.pdf]

Expanded View Figures

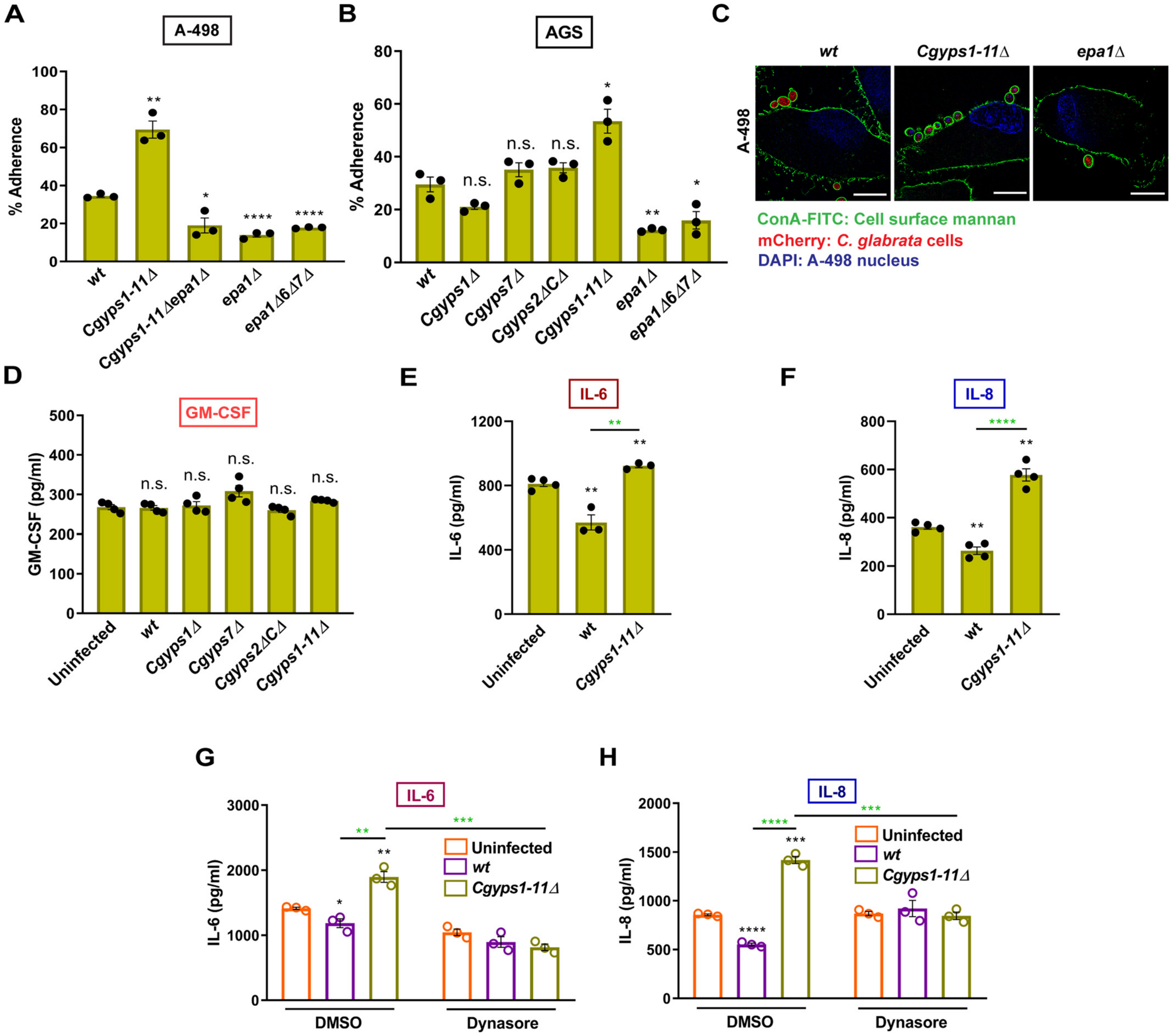

Figure EV1. *Cgyps1-11Δ* displays increased adherence to AGS cells.

(**A**) Adherence of indicated, S^{35}-labelled *Cg* strains to fixed A-498 (human kidney epithelial) cells after 2 h co-incubation. Black asterisks denote statistically-significant adherence differences in indicated strains, compared to *wild-type* (*wt*)-infected A-498. $n = 3$ biological replicates. (**B**) Adherence of indicated, S^{35}-labelled *Cg* strains to fixed AGS (human stomach epithelial) cells after 2 h co-incubation. Black asterisks denote statistically-significant adherence differences in indicated strain-infected, as compared to *wild-type* (*wt*)-infected AGS cells. $n = 3$ biological replicates. (**C**) Super resolution micrographs showing interaction of mCherry-expressing *wt*, *Cgyps1-11Δ* and *epa1Δ* strains with A-498 cells after 2 h co-culture. Infected epithelial cells were stained with concanavalin A (ConA)-FITC to differentiate between intracellular (ConA-non-stained) and extracellular (ConA-stained) *Cg*. DAPI was used to stain the host epithelial cell nuclei. Representative images were obtained from three biological experiments using Elyra 7 with 63X/1.44 NA objective lens. (**D**) Secreted GM-CSF (granulocyte-macrophage colony-stimulating factor) measurement in uninfected and *Cg*-infected A-498 cells after 24 h incubation. $n = 4$ biological replicates. (**E**, **F**) Secreted IL-6 (**E**) and IL-8 (**F**) levels in uninfected and *Cg*-infected A-498 cells. Infection was carried out at a MoI (multiplicity of infection) of 10:1. $n = 3$ biological replicates in (**E**), and $n = 4$ biological replicates in (**F**). (**G**, **H**) Secreted IL-6 (**G**) and IL-8 (**H**) levels in DMSO or dynasore (50 μM)-treated, *Cg*-infected, A-498 cells. Infection was carried out at 1:1 MoI. $n = 3$ biological replicates. Data information: In (**A**, **B**, **D–H**), data are presented as mean ± SEM. *$P < 0.05$; **$P < 0.01$; ***$P < 0.001$; ****$P < 0.0001$; n.s., not significant. Unpaired two-tailed Student's *t* test in (**A**, **B**, **D–H**). $P = 0.0016$ (*Cgyps1-11Δ* vs. *wt*), $P = 0.000072$ (*epa1Δ* vs. *wt*), $P = 0.0178$ (*Cgyps1-11Δepa1Δ* vs. *wt*), $P = 0.00002$ (*epa1Δ6Δ7Δ* vs. *wt*) in (**A**). $P = 0.0106$ (*Cgyps1-11Δ* vs. *wt*), $P = 0.0036$ (*epa1Δ* vs. *wt*), $P = 0.035$ (*epa1Δ6Δ7Δ* vs. *wt*) in (**B**). $P = 0.0031$ (*wt* vs. uninfected), $P = 0.0042$ (*Cgyps1-11Δ* vs. uninfected), $P = 0.002$ (*Cgyps1-11Δ* vs. *wt*) in (**E**). $P = 0.0016$ (*wt* vs. uninfected), $P = 0.0002$ (*Cgyps1-11Δ* vs. uninfected), $P = 0.00004128$ (*Cgyps1-11Δ* vs. *wt*) in (**F**). $P = 0.0306$ (*wt* vs. uninfected), $P = 0.0048$ (*Cgyps1-11Δ* vs. uninfected), $P = 0.0027$ (*Cgyps1-11Δ* vs. *wt*) $P = 0.0004$ (*Cgyps1-11Δ*-Dynasore vs. *Cgyps1-11Δ*-DMSO) in (**G**). $P = 0.00004918$ (*wt* vs. uninfected), $P = 0.0001$ (*Cgyps1-11Δ* vs. uninfected), $P = 0.00002526$ (*Cgyps1-11Δ* vs. *wt*), $P = 0.0004$ (*Cgyps1-11Δ*-Dynasore vs. *Cgyps1-11Δ*-DMSO) in (**H**). Scale bar = 10 μm in (**C**).

© The Author(s)

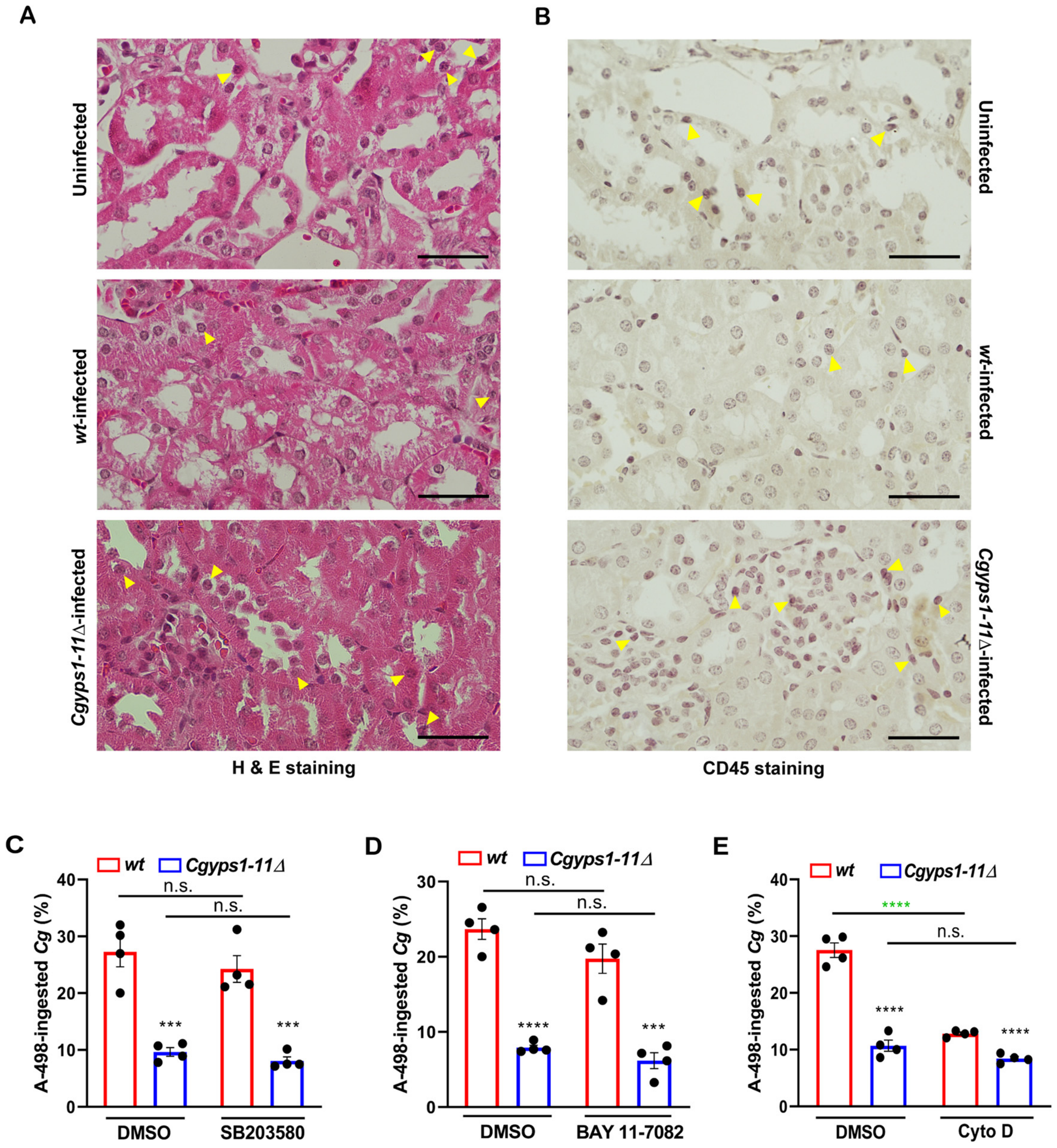

Figure EV2. p38 MAPK inhibition has no effect on *Cg* internalization.

(**A**, **B**) Micrographs of hematoxylin-eosin (H&E)-stained (**A**) and anti-CD45 antibody-stained (**B**) kidney tissue sections (40×) of uninfected, and *wt*- or *Cgyps1-11Δ*-infected mice at day 1 post-infection. $n = 3$ mice/group. Yellow arrowheads mark representative polymorphonuclear neutrophils infiltrated into the tissue. (**C**, **D**) CFU-based internalization analysis of *wt* and *Cgyps1-11Δ* strains in DMSO, SB203580 (10 μM; **C**) or BAY 11-7082 (10 μM; **D**) pre-treated A-498 cells, after 4 h co-incubation. The internalization percentage was calculated by dividing *Cg* CFUs recovered at 4 h by 0 h-CFUs (*Cg* cell number used for A-498 infection), and multiplying the number by 100. Asterisks mark differences between *wt* and *Cgyps1-11Δ* ingestion. $n = 4$ biological replicates. (**E**) CFU-based internalization analysis of *wt* and *Cgyps1-11Δ* strains in DMSO or cytochalasin D (Cyto D; 5 μM) pre-treated A-498 cells, after 4 h co-incubation. Black asterisks mark differences between *wt* and *Cgyps1-11Δ* ingestion. $n = 4$ biological replicates. Data information: In (**C–E**), data are presented as mean ± SEM. *$P < 0.05$; **$P < 0.01$; ***$P < 0.001$; ****$P < 0.0001$; n.s., not significant. Unpaired two-tailed Student's *t* test in (**C–E**). $P = 0.0007$ (*Cgyps1-11Δ*-DMSO vs. *wt*-DMSO), $P = 0.0006$ (*Cgyps1-11Δ*-SB203580 vs. *wt*-SB203580) in (**C**). $P = 0.000031$ (*Cgyps1-11Δ*-DMSO vs. *wt*-DMSO), $P = 0.0009$ (*Cgyps1-11Δ*-BAY 11-7082 vs. *wt*-BAY 11-7082) in (**D**). $P = 0.0000458$ (*Cgyps1-11Δ*-DMSO vs. *wt*-DMSO), $P = 0.0000942$ (*Cgyps1-11Δ*-CytoD vs. *wt*-CytoD), $P = 0.0000295$ (*wt*-CytoD vs. *wt*-DMSO) in (**E**). Scale bar = 20 μm in (**A**, **B**).

 © The Author(s)

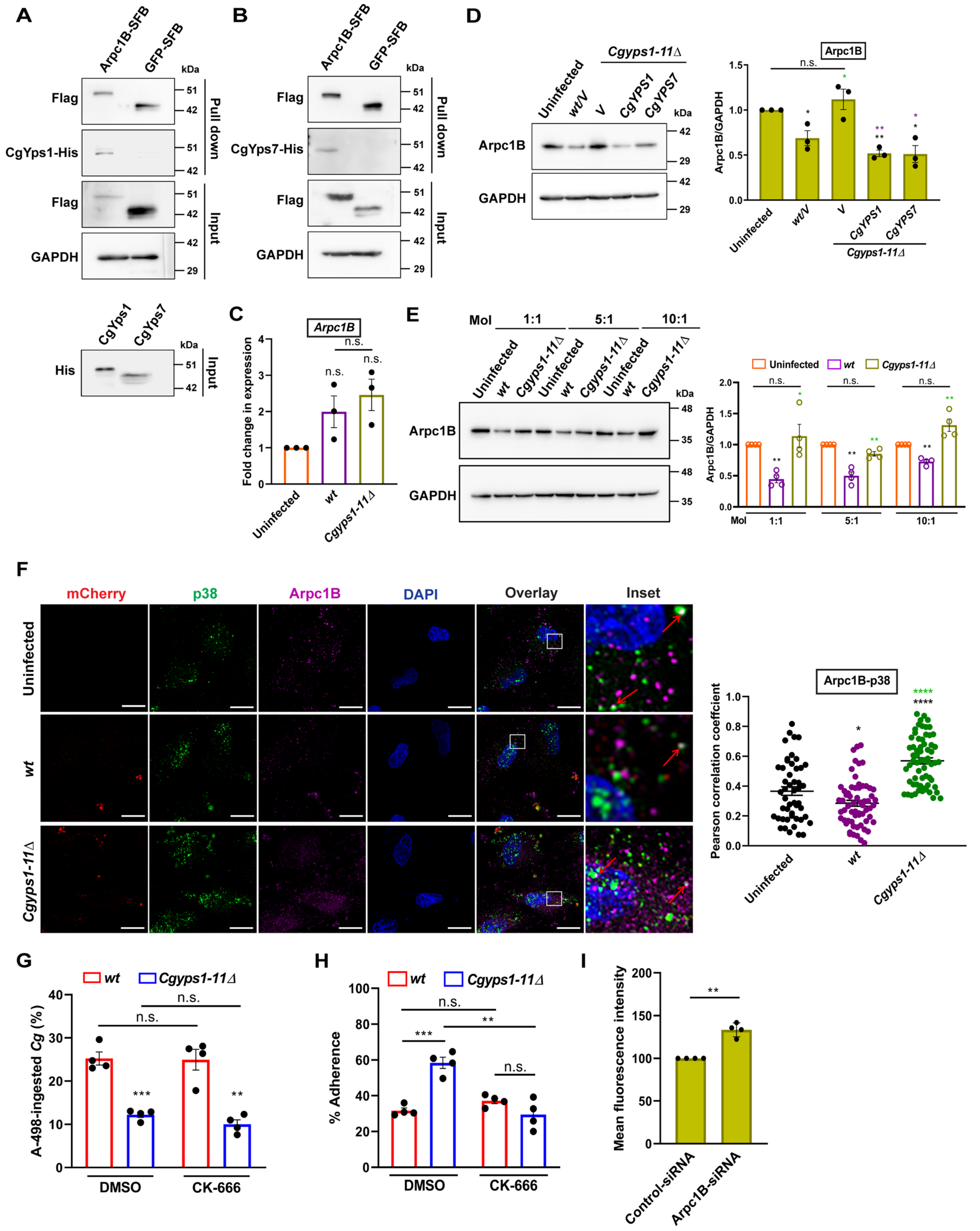

A
Arpc1B-SFB
GFP-SFB
kDa
Flag
CgYps1-His
Flag
GAPDH
Pull down
Input
CgYps1
CgYps7
His
B
CgYps7-His
C
Arpc1B
Fold change in expression
n.s.
Uninfected
wt
Cgyps1-11Δ
D
Cgyps1-11Δ
Uninfected
wt/V
V
CgYPS1
CgYPS7
Arpc1B
GAPDH
Arpc1B/GAPDH
E
MoI
1:1
5:1
10:1
Uninfected
F
mCherry
p38
Arpc1B
DAPI
Overlay
Inset
Arpc1B-p38
Pearson correlation coeffcient
G
A-498-ingested Cg (%)
DMSO
CK-666
H
% Adherence
I
Mean fluorescence intensity
Control-siRNA
Arpc1B-siRNA

© The Author(s)

Figure EV3. CgYps1 and CgYps7 interact with the epithelial cell protein Arpc1B.

(**A**, **B**) Representative immunoblots ($n = 2$ biological replicates) illustrating Arpc1B interaction with CgYps1 (**A**) and CgYps7 (**B**). Lysates (800 μg) of Arpc1B-SFB- or GFP-SFB-expressing A-498 cells were incubated with streptavidin beads, followed by incubation with 50-100 μg of *E. coli*-purified, 6X-histidine-tagged CgYps1 or CgYps7 protein. Input samples (*E. coli*-purified proteins; 30 μg) for CgYps1 (**A**) and CgYps7 (**B**) are shown underneath the blots of the panel (**A**). (**C**) qRT-PCR-based Arpc1B gene expression analysis in indicated A-498 cells after 6 h of *Cg* infection. Arpc1B gene expression was normalized against GAPDH mRNA control, and represent fold-change in Arpc1B transcript levels in *Cg*-infected, compared to uninfected A-498 cells (taken as 1.0). $n = 3$ biological replicates. (**D**, **E**) Representative immunoblots illustrating Arpc1B protein levels in A-498 cells that were left uninfected or infected for 6 h at 1:1 MoI with *wt* expressing empty plasmid (*V*) or *Cgyps1-11Δ* expressing *V*, *CgYPS1* or *CgYPS7* (**D**) or infected with *wt* and *Cgyps1-11Δ* strains at indicated MoI (**E**). The signal intensity in each lane was quantified using the ImageJ software, and Arpc1B levels were normalized against the corresponding GAPDH levels. Data are plotted on the right side of the blots. Purple, green and black asterisks indicate Arpc1B level differences, as compared to *Cgyps1-11Δ/V*-infected, *wt/V*-infected and uninfected A-498 cells, respectively. $n = 3$ biological replicates in (**D**), and $n = 4$ biological replicates in (**E**). (**F**) Representative confocal micrographs illustrating co-localization of Arpc1B and p38 MAPK in A-498 cells. A-498 cells were left uninfected or infected with mCherry-expressing *wt* and *Cgyps1-11Δ*-strains for 6 h, followed by labelling with anti-Arpc1B and anti-p38 antibodies. Cells from two biological infection experiments were imaged using the confocal microscope (Leica SP8) with 63X/1.44 NA objective lens in z-stack mode. Arpc1B and p38 signal intensities were measured for 50 foci in a minimum of 15 cells, using the LASX software, and their co-localization was determined via the Pearson correlation coefficient (PCC). Co-localization data are plotted on the right side of the micrographs. Arpc1B and p38 co-localization is indicated by red arrows in the Inset. Green and black asterisks indicate statistically-significant differences, compared to *wt*-infected and uninfected A-498 cells, respectively. (**G**) CFU-based internalization analysis of *wt* and *Cgyps1-11Δ* strains in DMSO or CK-666 (1 μM) pre-treated A-498 cells, after 4 h co-incubation. Asterisks mark differences between *wt* and *Cgyps1-11Δ* ingestion. $n = 4$ biological replicates. (**H**) CFU-based adherence analysis of *wt* and *Cgyps1-11Δ* strains in DMSO or CK-666 (1 μM) pre-treated A-498 cells, after 2 h co-incubation. $n = 4$ biological replicates. (**I**) Flow cytometry-based analysis of Annexin V staining in indicated A-498 cells. $n = 4$ biological replicates. Data information: In (**C–I**), data are presented as mean ± SEM. *$P < 0.05$; **$P < 0.01$; ***$P < 0.001$; ****$P < 0.0001$; n.s., not significant. Unpaired or paired two-tailed Student's *t* test in (**C–I**). $P = 0.0482$ (*wt/V* vs. uninfected), $P = 0.0375$ (*Cgyps1-11Δ/V* vs. *wt/V*), $P = 0.0056$ (*Cgyps1-11Δ/CgYPS1* vs. uninfected), $P = 0.0349$ (*Cgyps1-11Δ/CgYPS7* vs. uninfected), $P = 0.0073$ (*Cgyps1-11Δ/CgYPS1* vs. *Cgyps1-11Δ/V*), $P = 0.0145$ (*Cgyps1-11Δ/CgYPS7* vs. *Cgyps1-11Δ/V*) in (**D**). $P = 0.0025$ (*wt* at 1:1 MoI vs. uninfected), $P = 0.0129$ (*Cgyps1-11Δ* vs. *wt* at 1:1 MoI), $P = 0.004$ (*wt* at 5:1 MoI vs. uninfected), $P = 0.0026$ (*Cgyps1-11Δ* vs. *wt* at 5:1 MoI), $P = 0.0023$ (*wt* at 10:1 MoI vs. uninfected), $P = 0.0013$ (*Cgyps1-11Δ* vs. *wt* at 10:1 MoI) in (**E**). $P = 0.0178$ (*wt* vs. uninfected), $P = 0.00000004$ (*Cgyps1-11Δ* vs. uninfected), $P = 0.00000000000000001$ (*Cgyps1-11Δ* vs. *wt*) in (**F**). $P = 0.0002$ (*Cgyps1-11Δ*-DMSO vs. *wt*-DMSO), $P = 0.0013$ (*Cgyps1-11Δ*-CK-666 vs. *wt*-CK-666) in (**G**). $P = 0.0003$ (*Cgyps1-11Δ*-DMSO vs. *wt*-DMSO), $P = 0.0015$ (*Cgyps1-11Δ*-CK-666 vs. *Cgyps1-11Δ*-DMSO) in (**H**). $P = 0.0038$ (*Arpc1B*-siRNA vs. Control siRNA) in (**I**). Scale bar = 20 μm in (**F**).

 © The Author(s)

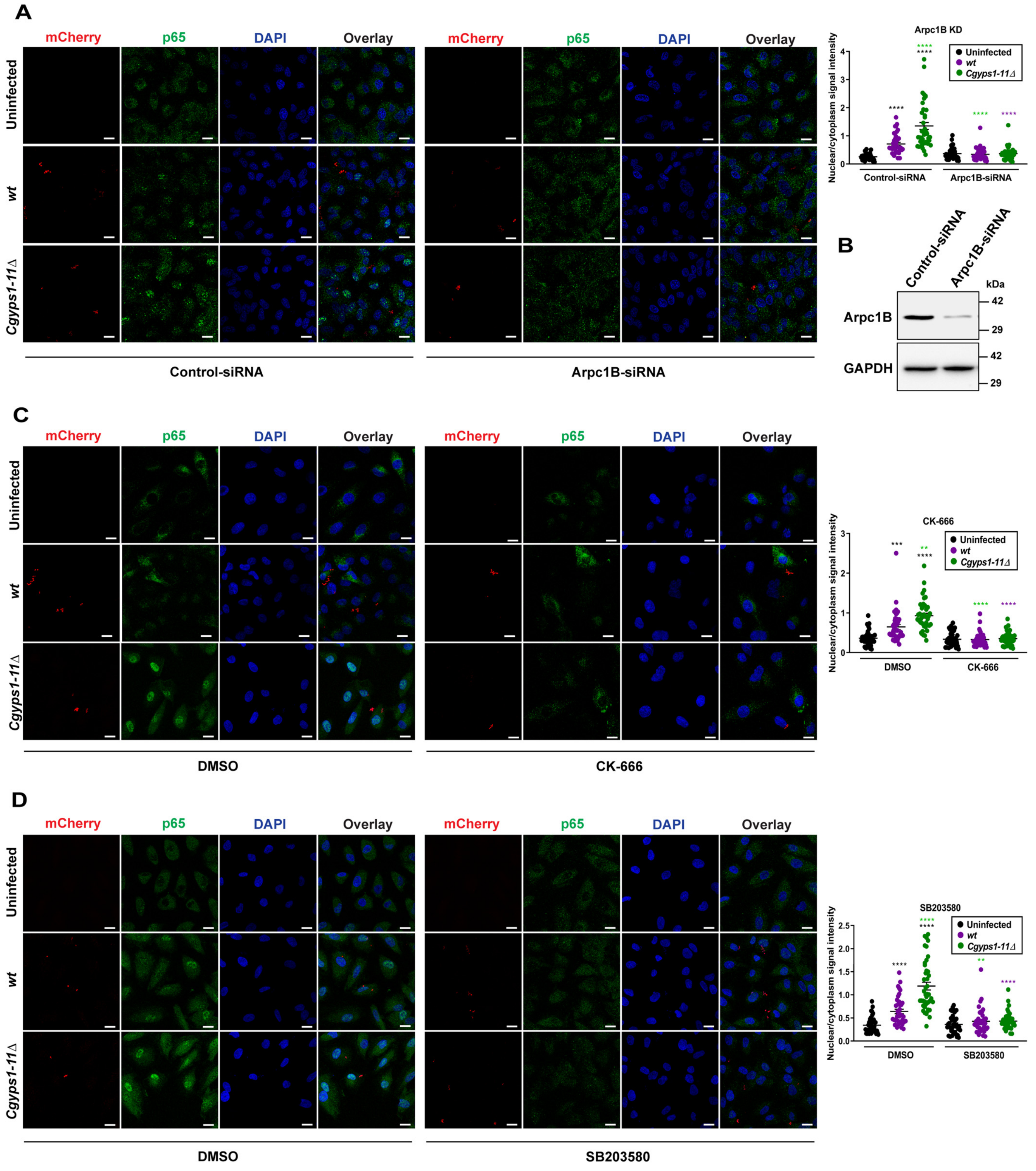
A
mCherry
p65
DAPI
Overlay
Uninfected
wt
Cgyps1-11Δ
Control-siRNA
Arpc1B-siRNA
Arpc1B KD
Nuclear/cytoplasm signal intensity
Uninfected
wt
Cgyps1-11Δ
B
Control-siRNA
Arpc1B-siRNA
kDa
Arpc1B
GAPDH
42
29
C
DMSO
CK-666
D
SB203580

Figure EV4. Arpc1B inhibition decreases p65 nuclear localization in *Cgyps1-11Δ*-infected A-498 cells.

(**A**) Confocal micrographs illustrating p65 cellular localization in control-siRNA or Arpc1B-siRNA treated A-498 cells, after 6 h of infection with mCherry-expressing *wt* or *Cgyps1-11Δ* cells. Immunofluorescence analysis of A-498 cells was performed with anti-p65 antibody, and images were captured using the confocal microscope (Leica SP8) with 63X/1.44 NA objective lens in z-stack mode. Overlay images show cytoplasmic and nuclear p65 distribution, with the anti-rabbit Alexa Flour-488 antibody in green, the mCherry-tagged *Cg* in red and the host cell nuclei in blue color. For quantification, fluorescence signal intensities in the cytoplasm and the nucleus were measured in a minimum of 40 cells in two biological experiments, using the ImageJ software. Data are plotted, as the ratio of nuclear to cytoplasm abundance, on the right side of micrographs. Green and black asterisks indicate statistically-significant differences, as compared to *wt*-infected and uninfected A-498 cells, respectively. (**B**) Representative immunoblots showing reduction in Arpc1B protein levels in A-498 cells that were transfected with Arpc1B-siRNA (10 μm), as compared to scrambled-siRNA (10 μm)-transfected A-498 cells. GAPDH was used as loading control. $n = 3$ biological replicates. (**C**, **D**) Confocal micrographs illustrating p65 cellular localization in DMSO or CK-666 (1 μM)-treated (**C**), and DMSO or SB203580 (10 μM)-treated (**D**) A-498 cells, after 6 h of infection with mCherry-expressing *wt* or *Cgyps1-11Δ* cells. Immunofluorescence analysis of A-498 cells was performed with anti-p65 antibody, and images were captured using the confocal microscope (Leica SP8) with 63X/1.44 NA objective lens in z-stack mode. Overlay images show cytoplasmic and nuclear p65 distribution, with the anti-rabbit Alexa Flour-488 antibody in green, the mCherry-tagged *Cg* in red and the host cell nuclei in blue color. For quantification, fluorescence signal intensities in the cytoplasm and the nucleus were measured in a minimum of 40 cells in two biological experiments, using the ImageJ software. Data are plotted, as the ratio of nuclear to cytoplasm abundance, on the right side of micrographs. Green and black asterisks indicate statistically-significant differences, as compared to *wt*-infected and uninfected A-498 cells, respectively. Data information: In (**A**, **C**, **D**), data are presented as mean ± SEM. **$P < 0.01$; ***$P < 0.001$; ****$P < 0.0001$; n.s. not significant. Unpaired two-tailed Student's *t* test in (**A**, **C**, **D**). $P = 0.0000000005$ (*wt*-Control siRNA vs. uninfected-Control siRNA), $P = 0.000000000001577$ (*Cgyps1-11Δ*-Control siRNA vs. uninfected-Control siRNA), $P = 0.0000234$ (*Cgyps1-11Δ*-Control siRNA vs. *wt*-Control siRNA), $P = 0.0000002043$ (*wt-Arpc1B* siRNA vs. *wt*-Control siRNA), $P = 0.0000000002$ (*Cgyps1-11Δ-Arpc1B* siRNA vs. *Cgyps1-11Δ*-Control siRNA) in (**A**). $P = 0.000143$ (*wt*-DMSO vs. uninfected-DMSO), $P = 0.000000000002$ (*Cgyps1-11Δ*-DMSO vs. uninfected-DMSO), $P = 0.0018$ (*Cgyps1-11Δ*-DMSO vs. *wt*-DMSO), $P = 0.00001608$ (*wt*-CK-666 vs. *wt*-DMSO), $P = 0.0000000000004$ (*Cgyps1-11Δ*-CK-666 vs. *Cgyps1-11Δ*-DMSO) in (**B**). $P = 0.00000104$ (*wt*-DMSO vs. uninfected-DMSO), $P = 0.000000000000023$ (*Cgyps1-11Δ*-DMSO vs. uninfected-DMSO), $P = 0.00000029$ (*Cgyps1-11Δ*-DMSO vs. *wt*-DMSO), $P = 0.0014$ (*wt*-SB203580 vs. *wt*-DMSO), $P = 0.000000000001$ (*Cgyps1-11Δ*-SB203580 vs. *Cgyps1-11Δ*-DMSO) in (**D**). Scale bar = 20 μm in (**A**, **C**, **D**).

 © The Author(s)

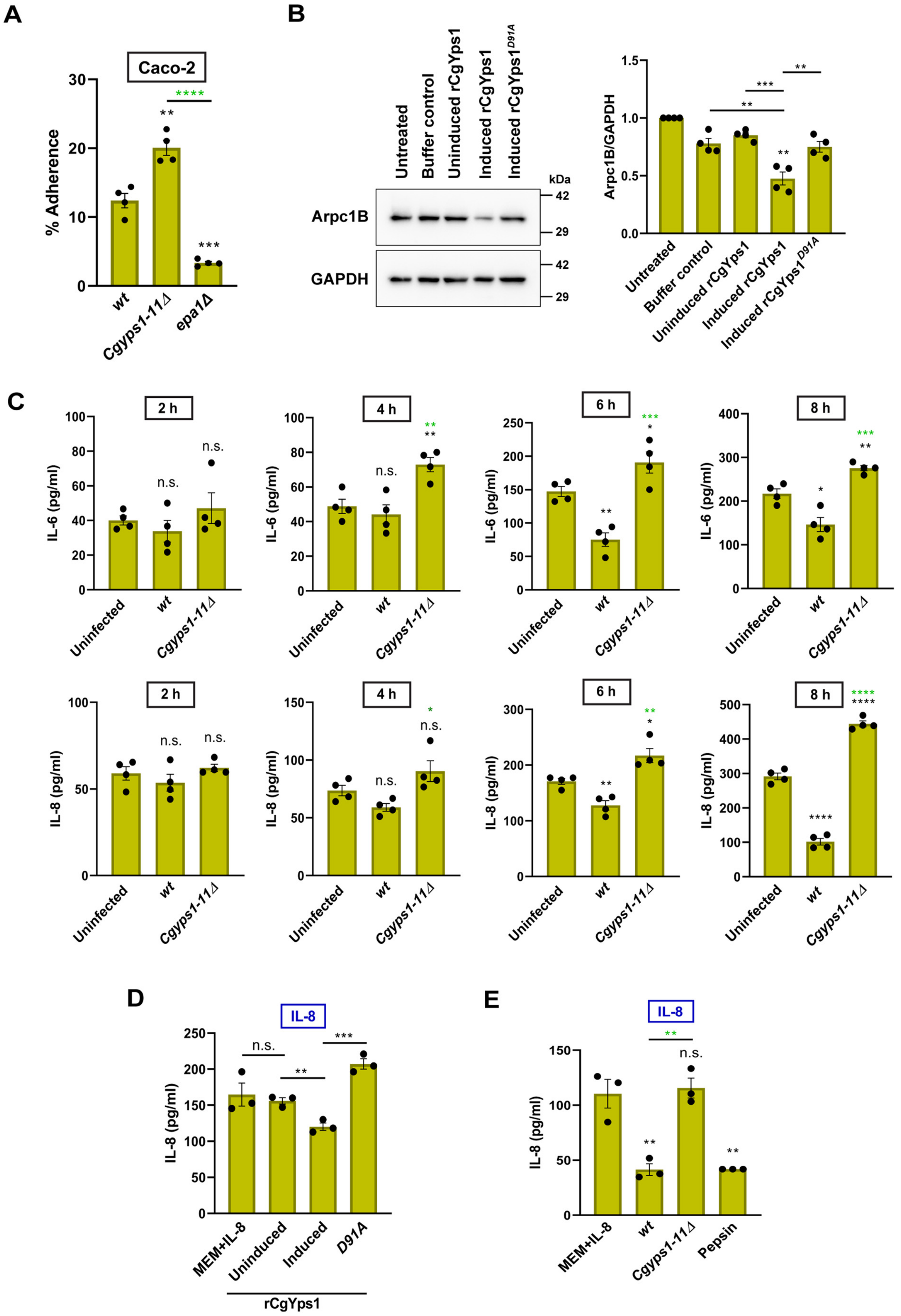
A
Caco-2
% Adherence
wt
Cgyps1-11Δ
epa1Δ
B
Untreated
Buffer control
Uninduced rCgYps1
Induced rCgYps1
Induced rCgYps1D91A
kDa
Arpc1B
GAPDH
Arpc1B/GAPDH
C
2 h
4 h
6 h
8 h
IL-6 (pg/ml)
IL-8 (pg/ml)
Uninfected
n.s.
D
IL-8
MEM+IL-8
Uninduced
Induced
D91A
rCgYps1
E
Pepsin

◀ **Figure EV5. rCgYps1 reduces Arpc1B levels.**

(**A**) Adherence of indicated *Cg* strains to fixed Caco-2 (human intestinal epithelial) cells after 2 h co-incubation, as determined by CFU-based assay. Black asterisks mark adherence differences, as compared to *wt*-coincubated A-498 cells. $n = 4$ biological replicates. (**B**) Representative immunoblots illustrating Arpc1B levels in A-498 cells that were either left untreated or incubated for 6 h with *P. pastoris*-partially purified rCgYps1 (100 µg), rCgYps1^{D91A} (100 µg), partially-purified proteins (100 µg) from uninduced supernatants of *P. pastoris* cells or citrate buffer (pH 6.0). rCgYps1 and rCgYps1^{D91A} protein expression in *P. pastoris* cells was induced by adding (2%) methanol. The signal intensity in each lane was measured using the ImageJ software, and Arpc1B levels were normalized against GAPDH levels. Data represent fold-change in Arpc1B levels in indicated conditions, as compared to untreated A-498 cells (considered as 1.0), and are plotted on the right side of the blots. $n = 4$ biological replicates. (**C**) Secreted IL-6 and IL-8 cytokine levels in uninfected, *wt*- and *Cgyps1-11Δ*-infected A-498 cells at indicated time points post-infection. Infection was carried at 5:1 MoI. Green and black asterisks indicate statistically-significant differences in cytokine secretion, as compared to *wt*-infected and uninfected A-498 cells, respectively. $n = 4$ biological replicates. (**D**) *P. pastoris*-partially-purified rCgYps1 and rCgYps1^{D91A} proteins (100 µg) were incubated with the human recombinant IL-8 cytokine (200 pg; MEM medium) for 4 h at 37 °C, and IL-8 levels were measured using the human IL-8 BD OptEIA ELISA kit. IL-8 incubated in MEM medium was used as control. $n = 3$ biological replicates. (**E**) Human recombinant IL-8 (100 pg) was incubated with overnight YPD-grown *wt* and *Cgyps1-11Δ* cells (1.0 $O.D_{600}$; MEM medium), pepsin (100 ng) or MEM medium for 6 h at 37 °C. Samples were centrifuged to remove *Cg* cells, and IL-8 levels in supernatants were measured using the human IL-8 BD OptEIA ELISA kit. The Pepsin enzyme was used as control. Black asterisks denote statistically-significant differences, as compared to IL-8 incubated in MEM medium. $n = 3$ biological replicates. Data information: In (**A–E**), data are presented as mean ± SEM. *$P < 0.05$; **$P < 0.01$; ***$P < 0.001$; ****$P < 0.0001$; n.s., not significant. Unpaired or paired two-tailed Student's *t* test in (**A–E**). $P = 0.0002$ (*epa1Δ* vs. *wt*), $P = 0.0024$ (*Cgyps1-11Δ* vs. *wt*), $P = 0.00000603$ (*Cgyps1-11Δ* vs. *epa1Δ*) in (**A**). $P = 0.003$ (Induced rCgYps1 vs. untreated), $P = 0.005$ (Induced rCgYps1 vs. buffer control), $P = 0.0008$ (Induced rCgYps1 vs. uninduced rCgYps1), $P = 0.009$ (Induced rCgYps1^{D91A} vs. induced rCgYps1) in (**B**). $P = 0.0058$ (*Cgyps1-11Δ*-4h vs. *wt*-4h), $P = 0.0063$ (*Cgyps1-11Δ*-4h vs. uninfected-4h), $P = 0.0013$ (*wt*-6h vs. uninfected-6h), $P = 0.0475$ (*Cgyps1-11Δ*-6h vs. uninfected-6h), $P = 0.0008$ (*Cgyps1-11Δ*-6h vs. *wt*-6h), $P = 0.0109$ (*wt*-8h vs. uninfected-8h), $P = 0.0037$ (*Cgyps1-11Δ*-8h vs. uninfected-8h), $P = 0.0003$ (*Cgyps1-11Δ*-8h vs. *wt*-8h) in IL-6 section of (**C**). $P = 0.0168$ (*Cgyps1-11Δ*-4h vs. *wt*-4h), $P = 0.0047$ (*wt*-6h vs. uninfected-6h), $P = 0.0153$ (*Cgyps1-11Δ*-6h vs. uninfected-6h), $P = 0.0011$ (*Cgyps1-11Δ*-6h vs. *wt*-6h), $P = 0.00000886$ (*wt*-8h vs. uninfected-8h), $P = 0.00002363$ (*Cgyps1-11Δ*-8h vs. uninfected-8h), $P = 0.00000019$ (*Cgyps1-11Δ*-8h vs. *wt*-8h) in IL-8 section of (**C**). $P = 0.0061$ (Induced rCgYps1 vs. uninduced rCgYps1), $P = 0.0006$ (Induced rCgYps1^{D91A} vs. induced rCgYps1) in (**D**). $P = 0.0079$ (*wt* vs. MEM + IL-8), $P = 0.0061$ (Pepsin vs. MEM + IL-8), $P = 0.0021$ (*Cgyps1-11Δ* vs. *wt*) in (**E**).

 © The Author(s)
